# Drug Bioavailability Enhancing Agents of Natural Origin (Bioenhancers) that Modulate Drug Membrane Permeation and Pre-Systemic Metabolism

**DOI:** 10.3390/pharmaceutics11010033

**Published:** 2019-01-16

**Authors:** Bianca Peterson, Morné Weyers, Jan H. Steenekamp, Johan D. Steyn, Chrisna Gouws, Josias H. Hamman

**Affiliations:** Centre of Excellence for Pharmaceutical Sciences (Pharmacen™), North-West University, Potchefstroom 2520, South Africa; bianca.peterson@nwu.ac.za (B.P.); weyers.morne@gmail.com (M.W.); jan.steenekamp@nwu.ac.za (J.H.S.); dewald.steyn@nwu.ac.za (J.D.S.); chrisna.gouws@nwu.ac.za (C.G.)

**Keywords:** bioenhancer, cytochrome P450, drug absorption enhancer, efflux, metabolism, P-glycoprotein, pharmacokinetic interaction, tight junction

## Abstract

Many new chemical entities are discovered with high therapeutic potential, however, many of these compounds exhibit unfavorable pharmacokinetic properties due to poor solubility and/or poor membrane permeation characteristics. The latter is mainly due to the lipid-like barrier imposed by epithelial mucosal layers, which have to be crossed by drug molecules in order to exert a therapeutic effect. Another barrier is the pre-systemic metabolic degradation of drug molecules, mainly by cytochrome P450 enzymes located in the intestinal enterocytes and liver hepatocytes. Although the nasal, buccal and pulmonary routes of administration avoid the first-pass effect, they are still dependent on absorption of drug molecules across the mucosal surfaces to achieve systemic drug delivery. Bioenhancers (drug absorption enhancers of natural origin) have been identified that can increase the quantity of unchanged drug that appears in the systemic blood circulation by means of modulating membrane permeation and/or pre-systemic metabolism. The aim of this paper is to provide an overview of natural bioenhancers and their main mechanisms of action for the nasal, buccal, pulmonary and oral routes of drug administration. Poorly bioavailable drugs such as large, hydrophilic therapeutics are often administered by injections. Bioenhancers may potentially be used to benefit patients by making systemic delivery of these poorly bioavailable drugs possible via alternative routes of administration (i.e., oral, nasal, buccal or pulmonary routes of administration) and may also reduce dosages of small molecular drugs and thereby reduce treatment costs.

## 1. Introduction

Drug absorption is the process whereby drug molecules are transferred from the site of administration across biological membranes into the systemic blood circulation to produce a systemic pharmacological effect. Biological cell membranes have a lipophilic nature due to their phospholipid bilayer structures. Molecules should therefore have sufficient hydrophilic properties to dissolve in the aqueous environments surrounding the biological membranes, but should also have sufficient lipophilic properties to partition into the membranes in order to achieve passive absorption via the transcellular pathway [1]. Adjacent epithelial/endothelial cells are connected by tight junctions, which are traversed by aqueous channels/fenestrae through which only small water-soluble molecules (<600 Da) can pass to get absorbed via the paracellular pathway [2].

A number of active transporter molecules (including both uptake transporters and efflux transporters) are present in various cell types in different organs. Drug efflux transporters found in the plasma membranes of intestinal epithelial cells can pump structurally diverse compounds from within the intestinal epithelial cells back to the gastro-intestinal lumen and thereby reduce drug bioavailability [3]. Efflux of compounds occurs by active transporters that need energy and this process is adenosine triphosphate (ATP)-dependent [4]. The ATP-binding cassette (ABC) transporter superfamily is among the largest and most broadly expressed efflux transporters discovered so far, consisting of P-glycoprotein (P-gp), the multidrug resistant protein (MRP) and the breast cancer resistance protein (BCRP) [5,6,7]. Another major determinant of oral drug bioavailability besides drug permeation across the epithelial cells is pre-systemic metabolism or first-pass metabolism, which is the metabolism that takes place during uptake before the drug molecules reach the systemic circulation, as observed for olanzapine treatment [8]. Pre-systemic metabolism occurs mainly in the enterocytes of the gastrointestinal epithelium and the hepatocytes of the liver. The cytochrome P450 (CYP) family of enzymes account for the majority of oxidative metabolic reactions of xenobiotics during pre-systemic and systemic metabolism. More than 30 different human CYP enzymes have been identified, of which CYP3A4 appears to be one of the most important drug-metabolizing enzymes in humans [9].

For the purpose of this paper, the term bioenhancer is reserved for molecules of natural origin that are capable of increasing the rate and/or extent at which co-administered drug molecules reach the systemic circulation unchanged (i.e., increased bioavailability). The main mechanisms that have been identified through which bioenhancers can improve the bioavailability of drug molecules include alteration of the plasma membrane fluidity to increase passive transcellular drug permeation; modulation of tight junctions to allow for increased paracellular diffusion; and active efflux transporter modulation, such as P-gp-related efflux inhibition. Inhibition of CYP enzymes in the intestinal epithelium and liver can significantly impact upon the bioavailability of drugs that are substrates of these enzymes by means of reducing pre-systemic metabolism [10,11,12].

The most popular route of drug administration remains the oral route [2]. As mentioned before, the oral bioavailability of a drug molecule is determined by its ability to penetrate the gastrointestinal epithelial membrane, which is mainly determined by its physico-chemical properties (e.g., pKa, lipophilicity, molecular size, charge, dissolution and solubility) [13], together with the extent of enzymatic metabolism during its movement to the systemic circulation (known as pre-systemic metabolism or the first-pass effect). Some other factors that may affect the oral bioavailability of a drug include the gastric emptying rate, pH of the gastrointestinal fluid, interactions with other compounds (e.g., other drugs, food or herbs) and its affinity for active transporters [2,14].

Drug administration via the nasal route can easily be accomplished by patients for both local and systemic drug delivery, which is non-invasive and painless. A relatively large epithelial surface is available that is highly permeable and offers a rapid onset of therapeutic effect. For drugs that target the central nervous system, direct nose-to-brain drug delivery is possible [13,15,16]. Additionally, intranasal drug administration bypasses hepatic first-pass metabolism [13,17,18]. However, the protective mucous layer and ciliary clearance may potentially have a negative impact on intranasal absorption [13,15].

The buccal route of administration is a good alternative for drugs that are unstable in gastric fluids and those that are severely affected by first-pass metabolism. However, absorption across the buccal mucosa is relatively slow due to the limited surface area, poor permeability of buccal epithelial tissue, removal of drug by saliva and the presence of peptidases within the buccal mucosa [15]. Hence this route of drug administration is mostly suited for highly potent, low dose drugs [15].

The pulmonary route of drug administration (i.e., administration via the lungs) is associated with rapid drug delivery due to the large surface area and abundant blood supply [2,15]. Pulmonary drug delivery can occur through different dosage forms, such as aerosol or nebulizer, for both local treatment (e.g., bronchodilators) or systemic drug delivery [2]. In the case of volatile anesthetics or for voluptuary drugs, the inhalation route is the preferred way of administration [2,19].

In this paper, discussions regarding drug absorption enhancing agents are restricted to bioenhancers of natural origin (therefore purely synthetic chemical permeation enhancers are excluded). The selected bioenhancers are discussed in terms of their main effects on drug bioavailability as well as their mechanisms of action as elucidated by in vitro and in vivo studies. Furthermore, a comprehensive list of bioenhancers is included in Table 1 for each of the four selected routes of administration including the buccal, nasal, pulmonary and oral routes of drug administration.

## 2. Buccal Route of Administration

**Figure 1:**
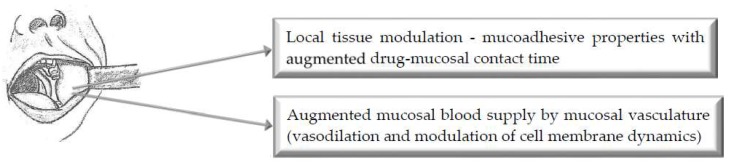
Illustration of the main mechanisms of action of bioenhancers for enhanced buccal drug delivery.

Figure 1 illustrates the main mechanisms of action of selected bioenhancers for improved drug delivery via the buccal route of administration.

### 2.1. Aloe Vera

The effect of *Aloe vera* gel on the permeability of didanosine (ddI) across porcine buccal mucosae was investigated using Franz diffusion cells. The control solution contained ddI in phosphate buffer saline (PBS) at pH 7.4 alone (5, 10, 15, 20 mg/mL), and the test solutions contained ddI (20 mg/mL) in the presence of *A. vera* gel (0.25, 0.5, 1, 2, 4, and 6% *w/v*) [20].

At concentrations of 0.25 to 2% *w/v*, *A. vera* gel significantly enhanced the buccal permeability of ddI with enhancement ratios ranging from 5.09 (0.25% *w/v*) to 11.78 (2% *w/v*). However, at higher concentrations (4 and 6% *w/v*) of *A. vera* gel, decreased ddI permeability across the buccal tissue was observed. This may be attributed to the high viscosity of the *A. vera* gel at these high concentrations, which caused resistance to drug diffusion. *A. vera* gel may be used as a potential buccal permeation enhancer for ddI in the treatment of HIV and AIDS [20].

### 2.2. Bile Salts

The in vitro permeation of 2′,3′-dideoxycytidine (ddC) across porcine buccal mucosae was studied in the absence and presence of sodium glycocholate using in-line flow-through diffusion cells [28]. Fresh isotonic McIlvaine buffer solution (IMB, pH 7.4), which simulated gingival fluid without enzyme, with 10 mg/mL ddC, 0.01% (*w/v*) gentamicin and sodium glycocholate in concentrations varying from 0.6 to 50 mM, were added to the donor chambers. A flow rate of 0.8 mL/h was maintained, and samples were collected every 90 min for 22.5 h. Results demonstrated a concentration-dependent increase in ddC permeation across buccal mucosa as donor concentrations of ddC was increased from 1 to 20 mg/mL. This is indicative of passive diffusion [28]. In the presence of sodium glycocholate (4 mM), the permeability of ddC was significantly increased (~32-fold) to an apparent permeability coefficient (P_app_) value of 5.11 ± 1.46 × 10^−6^ cm/s. At lower sodium glycocholate concentrations (<4 mM), a limited enhancement effect was observed, while the P_app_ value was only increased to 5.61 ± 1.06 × 10^−6^ cm/s at higher concentrations of sodium glycocholate (10 and 50 mM). Earlier studies indicated that 4 mM sodium glycocholate was close to the critical micelle concentration (CMC) of sodium glycocholate [116,117]. Since sodium glycocholate can solubilize the membrane lipids by incorporating them into sodium glycocholate micelles, a low enhancement effect was expected at sodium glycocholate concentrations below the CMC of 4 mM. On the other hand, interfacial saturation between sodium glycocholate micelles and lipid could explain the restricted enhancement effect observed with sodium glycocholate at concentrations beyond the CMC [28].

### 2.3. Chitosan and Derivatives

It was shown that chitosan could enhance the absorption of the transforming growth factor-β (TGF-β), a large bioactive peptide, across buccal mucosal tissue. A gel was prepared that consisted of 2% chitosan-H (MW: 1 400,000; degree of deacetylation: 80%) in dilute lactic acid solution. I125-labelled TGF-b (MW: ~25 Kda) was incorporated into the chitosan gel, as well as in a control solution of PBS. Continuous-flow perfusion chambers were used to study the permeability of TGF-β across porcine buccal mucosa dermatomed to a thickness of approximately 700 µm. Additionally, horizontal sectioning and counting was performed to determine the localization of TGF-β within the buccal mucosa [22]. Results demonstrated that chitosan enhanced the permeability of the TGF-β bioactive peptide in buccal mucosa six- to seven-fold, even though oral mucosa is relatively impermeable to TGF-β due to its large size [22].

Furthermore, compared to the control PBS solution, an increased amount of TGF-β was found in the superficial layers of the epithelium [22]. Enhanced penetration of TGF-β into buccal mucosa may be the result of increased retention of the drug at the application site due to the mucoadhesive nature of chitosan [22]. Another potential mechanism whereby chitosan improved drug transport across the buccal epithelium, is interference with the lipid organization in the intercellular regions of the epithelium [22].

Another in vitro study demonstrated decreased trans-epithelial electrical resistance (TEER) of the buccal epithelial TR146 cell culture model when chitosan was used as a bioenhancer for peptide and protein absorption. In this study, chitosan glutamate concentrations of 20 µg/mL and higher showed enhanced transport of large hydrophilic compounds, ^3^H-mannitol and fluorescein isothiocyanate labeled dextrans (FITC–dextrans), at pH 6. ^3^H-mannitol demonstrated the highest cellular permeability, and decreasing permeability was observed for FITC–dextran with molecular weight (MW) of 4000 Da (FD4), FD10 (MW of 10,000 Da), and FD20 (MW of 20,000 Da) as their molecular weights increased [21]. Enhanced permeability caused by chitosan of all the test substances, except FD20, was statistically significant. Compared to untreated cells, the TEER of the TR146 cell culture model was drastically reduced to ~30% in the presence of chitosan glutamate at concentrations of 20 µg/mL and higher [21]. Contrary to nasal and intestinal mucosal membranes, the buccal mucosal intercellular barrier is not based on tight junctions [22], therefore tight junction modulation cannot be the mechanism by which permeability enhancement proceeded. It was thus suggested that interference with the lipid organization in the buccal mucosa was responsible for improved drug transport in the presence of chitosan glutamate or also potential loosening of intercellular filaments [21].

Thiolated chitosans have shown the ability to significantly improve mucoadhesion and drug permeation [118]. A mucoadhesive buccal peptide drug delivery system with thiolated chitosan was designed and evaluated in vitro and in vivo as an approach for the buccal delivery of pituitary adenylate cyclase-activating polypeptide (PACAP) [23,24]. Chitosan-4-thiobutylamidine (chitosan–TBA) was synthesized and homogenized with enzyme inhibitor and permeation mediator glutathione (GSH), lyophilized and compressed into flat-faced discs. Two experimental formulations were prepared for in vivo evaluation, namely, formulation A that comprised chitosan–TBA (69.5 mg), GSH (3.75 mg), Brij 35 (0.75 mg), and PACAP (1 mg); whereas formulation B contained an innermost layer of chitosan–TBA (50 mg), GSH (2.5 mg), Brij 35 (2.5 mg), PACAP (1 mg) and an outermost layer of chitosan–TBA (50 mg). Additionally, formulation B contained a palm wax coating on one side to ensure unidirectional release of the drug toward buccal mucosa, whilst avoiding losses in the oral cavity [23]. Control formulations contained unmodified chitosan and PACAP (formulation C) or unmodified chitosan, Brij 35, and PACAP (formulation D). These test formulations were given to pigs via buccal administration for 6 h. An absolute bioavailability of 1% was obtained with formulations A and B, whereas the controls (formulations C and D) did not allow PACAP to even reach the systemic circulation [23].

In another study, trimethyl chitosan (TMC) with different quaternization degrees (QDs of 4%, 35% and 90%) showed increased mucoadhesive properties and enhanced permeation of FD4 (FITC–dextran with a molecular weight (MW) of 4000 Da) across porcine cheek mucosa [29]. The epithelium of the porcine cheek mucosa was peeled from the underlying tissues and mounted in Franz diffusion cells. TMC polymer solutions were prepared at 4% (*w/w*) concentration by gentle stirring at room temperature, followed by addition of FD4 at 0.2% (*w/w*) concentration [29]. A tensile stress tester was used to evaluate the mucoadhesive properties of the polymer solutions on cheek buccal mucosa and submaxillary bovine mucin. Results from the in vitro permeation studies demonstrated that permeation of FD4 across the excised cheek mucosal tissue was poor and difficult in the absence of a penetration enhancer. Mucoadhesive performance increased with increasing QD, regardless of media or biological substrate used. However, increased permeation of FD4 was only observed with pH 6.4 buffer, which can be attributed to increased polymer solubility. The best permeation enhancement results were obtained with low molecular weight TMCs with high QD [29].

### 2.4. Fatty Acids

A study investigated the effect of unsaturated fatty acids (including oleic acid, eicosapentaenoic acid (EPA), and docosahexaenoic acid (DHA)) when concomitantly administered with insulin in a Pluronic F-127 (PF-127) gel formulation [27]. PF-127 (MW: 12,500 Da) was used to prepare insulin gel formulations with or without unsaturated fatty acids. The final concentrations of PF-127 and unsaturated fatty acids were 20 and 5%, respectively. The control formulation consisted of a 20% PF-127 gel containing only insulin [27]. In vitro release studies were performed using membrane-less dissolution tests. In vivo studies were performed by buccally administering a volume of 0.2 mL of a formulation (insulin dose, 25 IU/kg) to anesthetized rats. Blood samples were taken before and after buccal dosing to determine the serum glucose levels [27].

A decreased rate of insulin release together with a remarkable and continuous hypoglycemic effect was observed with PF-127 gels (insulin dose, 25 IU/kg) containing unsaturated fatty acids. The reduced release rate may be partly due to reductions in the numbers and dimensions of the aqueous channels through which the hydrophilic solutes diffuse, or it may be due to the viscosity of the formulations [27]. The serum glucose levels were significantly reduced with all formulations containing unsaturated fatty acids. Results demonstrated that PF-127 gels containing oleic acid yielded the highest bioavailability (15.9 ± 7.9%) of insulin relative to subcutaneous administration. In comparison, EPA and DHA yielded bioavailabilities of 3.4 ± 1.2% and 4.1 ± 3.4%, respectively. However, increased bioavailability observed in the presence of DHA was not statistically significant [27].

Similarly, a study investigated the effect of cod-liver oil extract (CLOE) and hydrogenated castor oil (HCO) on the buccal permeation of ergotamine tartrate (ET) [25]. Hamster cheek pouch was used as a model membrane for the in vitro permeation study using a two-chamber diffusion cell (37 °C). The buccal membrane was pre-treated for 1 or 3 h with a solution of phosphate buffer (PB, pH 7.4) and propylene glycol (PG) (PB:PG, 1:1) containing 5% of each of the permeation enhancers which was each added to the donor cell. After pre-treatment, the solutions in both cells were removed and the cells were rinsed multiple times using a fresh PG/PB mixture. This was immediately followed by the permeation experiment where the donor cell was filled with a suspension of ET in PG/PB, and the receiver cell was filled with the PG/PB mixture only [25]. Results from the permeation study demonstrated that the permeation rate of ET was markedly increased in the presence of each of the permeation enhancers (5%). In the presence of HCO, the solubility of ET was noticeably increased, which resulted in a relatively low flux of ET due to a decrease in the partitioning of ET to the mucosa [25]. On the other hand, the solubility of ET increased ~2-fold in the presence of CLOE, which produced a ~8-fold increase in the flux of ET. These results suggest that CLOE has a direct action on the mucosa in addition to the solubilizing effect it has on ET [25]. Increasing concentrations of CLOE did not enhance the permeation of ET greatly, and 3% concentration of CLOE was considered to be sufficient to exhibit the enhancing action. Furthermore, the flux of ET was almost constant with or without pre-treatment, and an extended period of pre-treatment had no effect on the flux of ET. These findings suggest that CLOE exhibits a transient enhancing effect [25].

The permeation study was repeated on four of the major fatty acids in CLOE, namely palmitic acid, oleic acid, eicosapentaenoic acid (EPA), and docosahexaenoic acid (DHA). However, the concentration of each fatty acid in the donor solution was calculated according to its composition ratios in CLOE. Results from this permeation study revealed that the individual fatty acids had a significantly lower effect on the flux of ET than that of 5% CLOE, of which oleic acid showed the greatest enhancing action. This suggests that the synergistic action of the individual fatty acids in CLOE likely contributes to the greater enhancing action of CLOE [25].

### 2.5. Menthol

A study of the trans-buccal permeation of dideoxycytidine (ddC) using menthol as an enhancer demonstrated a significant increase in ddC permeability. Porcine buccal tissue was used for the permeation experiments using side-bi-side flow-through diffusion cells at 37 °C. The P_app_ of ddC across the buccal mucosa increased 2.02 times at a menthol concentration of 0.3 mg/mL. However, no significant difference was observed between the permeation enhancement of ddC in the presence of lower concentrations (0.1 and 0.2 mg/mL) of l-menthol. This may be due to the limited effect of menthol on the intercellular lipid extraction over the range of concentrations studied [26]. It was suggested that the observed enhancement in ddC permeation may be partly due to the partition coefficient enhancing effects of l-menthol [26].

## 3. Nasal Route of Administration

**Figure 2:**
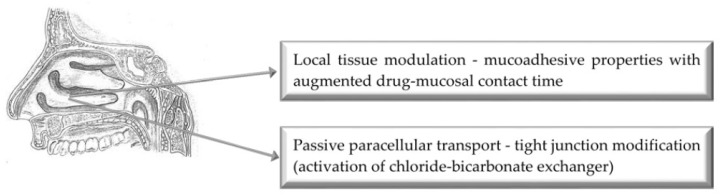
Illustration of the main mechanisms of action of bioenhancers for enhanced nasal drug delivery.

Figure 2 illustrates the main mechanisms of action of selected bioenhancers for improved drug delivery via the nasal route of administration.

### 3.1. Bile Salts

The bioavailability of insulin from a nasal formulation with 5% glycofurol (GF) was studied in rabbits [119]. Zinc-free human insulin, Novolin^®^ Nasal (Novo Nordisc) and glycofurol 75 (Hoffman La-Roche) were used in this study. Preparation of intranasal formulations consisted of 6.6 mg insulin dissolved in 1 mL phosphate buffer (12.5 mM, pH 7.4) containing 5% glycofurol. The dosage administered was 50 μL of the nasal solution within each nostril equivalent to 0.66 mg insulin (15.8 IU). Blood glucose was determined in blood collected at pre-determined time intervals. The frog palate model described by Gizurarson, et al. [120] was used to test local toxicity on mucocilliary clearance with phosphate buffer (12.5 mM) at pH 7.4 and 5% glycofurol. The results demonstrated greater decreased plasma glucose levels when glycofurol was included in the nasal formulation, which were comparable with previously published results [121]. Although the absorption enhancing mechanism of glycofurol is not known, the results demonstrated rapid insulin absorption with 90% reduction in the initial glucose blood concentration after 15 min. Furthermore, the plasma glucose was still suppressed with 85% of the initial value at a time interval of 120 min. The results are indicative of glycofurol having the ability to enhance insulin absorption in rats with enduring suppression of blood glucose levels.

Bagger, et al. [122] investigated the absolute nasal bioavailability of peptide T in rabbits when administered with sodium glycocholate and glycofurol. Previous nasal absorption studies with the bile salt sodium glycocholate as a drug absorption enhancer of peptide and peptide-like compounds has shown apparent bioenhancing effects [123,124,125,126,127]. In this study, the absorption enhancing action of sodium glycocholate and glycofurol was investigated by using *T*_max_, *C*_max_ and time-dependent concentration profiles of peptide T. The bioavailability of peptide T administered with the selected bioenhancers was compared to a control formulation consisting of peptide T in water, which gave a bioavailability of 5.9%. Sodium glycocholate showed the highest increase in the bioavailability of peptide T (59%), while glycofurol also increased its bioavailability (22%). On the other hand, when the two bioenhancers were combined (i.e., glycoferol and sodium glycocholate), a bioavailability of 29% was obtained for peptide T. Furthermore, when peptide T was co-administered with sodium glycocholate, the bioenhancing effect was characterized by rapid absorption but relatively short duration of action. The glycofurol showed a lower absorption enhancement effect, but with a longer duration of action [122].

### 3.2. Chitosan and Derivatives

Chitosan is a polysaccharide obtained from deacetylation of chitin, the second most abundant natural polymer that is contained in the exoskeletons of insects and crustaceans. Illum and co-workers were the first to demonstrate the nasal drug absorption enhancement effects of chitosan. Chitosan showed, for example, the ability to increase the *C*_max_ of insulin in sheep from 34 mIU/L to 191 mIU/L, while the AUC was elevated 7-fold [128]. The use of chitosan as a novel absorption enhancer for peptide drugs has been described previously [129], while its use in nasal delivery systems for a range of therapeutics has recently been comprehensively reviewed [130]. Selected studies will briefly be discussed below as illustration of the nasal drug delivery enhancement potential of the natural polymer, chitosan.

Illum, Watts, Fisher, Hinchcliffe, Norbury, Jabbal–Gill, Nankervis and Davis [31] showed that the co-administration of either chitosan in solution or chitosan formulated in microspheres can improve the bioavailability of morphine hydrochloride after nasal administration in sheep. Chitosan caused an increase in the *C*_max_ of morphine from 151 nM in the control group (morphine alone) to 657 nM, and improved the bioavailability of morphine from 10.0% (control group) to 26.6%. Furthermore, the rate of absorption was increased as indicated by the *T*_max_ of 14 min as opposed to 20 min in the control group. Chitosan formulated into microspheres even further improved the bioavailability parameters of morphine with a *C*_max_ of 1,010 nM and bioavailability of 54.65%. The rate of absorption was also improved with a *T*_max_ of 8 min, which was statistically significantly different from that of the control [128].

Hinchcliffe, Jabbal–Gill and Smith [30] investigated the pharmacokinetic effects of a chitosan-based intranasal delivery system on salmon calcitonin within a sheep model. A control nasal solution containing salmon calcitonin (2200 IU/mL) only was compared to a salmon calcitonin solution containing chitosan glutamate (5 mg/mL) as well as to Miacalcin^®^, a commercially available salmon calcitonin containing nasal spray. A *C*_max_ value of 99 pg/mL (range 50–107 pg/mL) was obtained for the salmon calcitonin solution containing chitosan compared to 33 pg/mL (range 13–49 pg/mL) for the salmon calcitonin control solution and 42 pg/mL (range 15–79 pg/mL) for the Miacalcin^®^ nasal spray. Furthermore, the average AUC value of the chitosan-containing solution of 3220 pg/mL/min (range 1606–4972 pg/mL/min) demonstrated a 3.5-fold increase compared to that of the control salmon calcitonin solution (943 pg/mL/min, range 198–2519 pg/mL/min) and 2-fold increase towards that of the Miacalcin^®^ nasal spray (1636 pg/mL/min, range 87–3792 pg/mL/min) [30].

Since chitosan is only soluble at acidic pH values below its pKa value, chemically modified derivatives, such as N-trimethyl chitosan chloride (TMC), have been synthesized to improve solubility at more neutral pH values [131]. In a study where TMC polymers with different quaternisation degrees (QD) was nasally administered with ^14^C-mannitol to rats, it was shown that the QD of TMC played an important role at a neutral environment (pH 7.4) in terms of its nasal absorption enhancement effects. The nasal delivery of ^14^C-mannitol increased with an increase in QD until a threshold value was reached at 45% [33].

### 3.3. Starch Microspheres

In vivo studies in sheep found augmented nasal drug absorption for bioadhesive starch microsphere delivery systems, which could be improved synergistically by combination with other absorption enhancing agents. The comparison of control solutions with starch microspheres containing insulin and gentamicin showed 5-fold and 10-fold increases in absorption efficiency, respectively [132,133,134]. Similar starch microsphere formulations and drug compounds demonstrated approximately 30-fold increases in the bioavailability of the drugs when administered nasally to rats [135].

## 4. Oral Route of Administration

**Figure 3:**
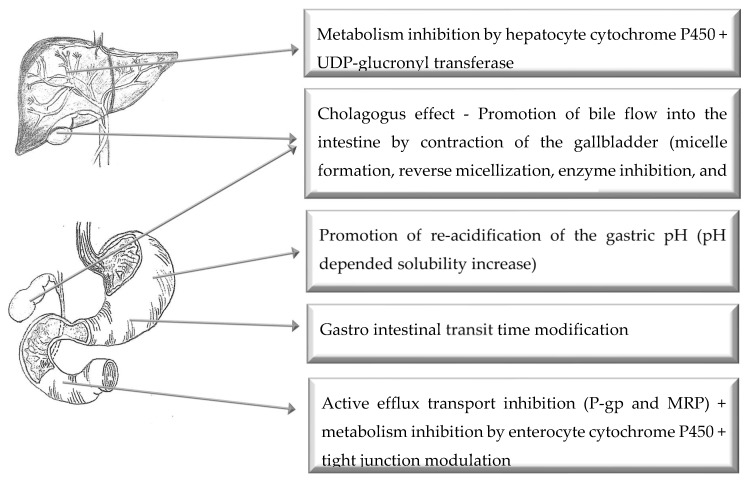
Illustration of the main mechanisms of action of bioenhancers for enhanced oral drug delivery.

Figure 3 illustrates the main mechanisms of action of selected bioenhancers for improved drug delivery via the oral route of administration.

### 4.1. Aloe Vera

*Aloe vera* leaf materials and extracts have been found to modify in vitro drug transport and in vivo drug bioavailability. In a double-blind, cross-over clinical study investigating the effect of *A. vera* liquid products on the absorption of vitamins C and E in human subjects, both *A. vera* gel product (AVG) and *A. vera* whole leaf product (AVWL) were investigated. AVG caused a 3.7-fold and AVWL a 2-fold increase in the bioavailability of vitamin C in comparison to the control (i.e., vitamin C administered with water). With respect to the influence on the bioavailability of vitamin E, both *Aloe* products caused a statistically significant increase in the baseline levels of vitamin E at 6 and 8 h post administration. However, due to large inter-individual variation, the AUC values between the different treatments were not statistically significant. The authors attributed the improvement in the bioavailability of vitamins C and E by the *A. vera* products to a protective action against degradation in the gastrointestinal tract, however, this was not proven in the study [37].

Both *A. vera* gel and whole leaf materials increased insulin transport extensively across Caco-2 cell monolayers over a concentration range of 0.1 to 5% *w/v* at two different pH values of 5.8 and 7.4. The *Aloe* materials decreased the TEER of the Caco-2 cell monolayers markedly at concentrations higher than 0.5% *w/v*, which was reversible [36]. A study was conducted using *A. ferox* and *A. vera* gel material, whole leaf material as well as precipitated polysaccharides (from these materials) in a concentration of 2% *w/v* in combination with atenolol as model drug across excised rat intestinal tissues in diffusion chambers. All the *Aloe* materials lowered the TEER of the excised rat intestinal tissues statistically significantly (*p* < 0.05) in comparison to the control (atenolol alone) and to a larger extent than the positive control (0.2% *w/v* sodium lauryl sulfate). In this study, it was also shown that some precipitated polysaccharides resulted in a higher decrease in TEER than their corresponding gel and whole leaf material counterparts. This reduction in TEER was indicative of the ability of the *Aloe* leaf materials to open tight junctions and consequently enhance paracellular transport of hydrophilic drug molecules such as atenolol. Only the precipitated polysaccharide fraction from dehydrated *A. vera* gel (Daltonmax 700^®^) material could enhance the transport of atenolol across intestinal rat tissue statistically significantly in comparison to the control. Although not statistically significant in comparison to the control, the polysaccharide fraction obtained from the *A. vera* whole leaf extract (Daltonmax 700^®^) caused a substantial increase in atenolol transport. The *A. ferox* materials were, however, not able to enhance the transport of atenolol across the excised rat intestinal tissues [35].

In a study by Wallis, Malan, Gouws, Steyn, Ellis, Abay, Wiesner, P Otto and Hamman [38], the effect of *A. vera* gel and polysaccharides (i.e., crude polysaccharides as well as fractionated polysaccharides based on molecular weight) on the bioavailability of indinavir was investigated in Sprague–Dawley rats. As part of this study, the effect of the selected *Aloe* materials was also investigated on the TEER of Caco-2 cell monolayers as well as their influence on the metabolism of indinavir in LS 180 cells. The results of this study indicated that all of the *Aloe* materials decreased the TEER of the Caco-2 cell monolayers indicating opening of tight junctions. The precipitated polysaccharides decreased the TEER to a larger extent than the gel material. However, no clear correlation between the different molecular weight fractions of the polysaccharides and TEER reduction could be made. All of the *Aloe* materials exhibited enzyme inhibitory effects, although not statistically significant, in comparison to the control (indinavir alone). With respect to the influence on the bioavailability of indinavir as indicated by AUC, all the investigated *Aloe* materials rendered an increase in bioavailability although not statistically significant. The increase in indinavir bioavailability caused by the crude precipitated polysaccharide and polysaccharide fractions were higher than that seen for the *A. vera* gel. The increase in bioavailability of indinavir in this study was attributed to a combination of mechanisms including the opening of the tight junctions (as indicated by a reduction in TEER) and an inhibition in the metabolism of indinavir (as indicated by the metabolite plasma concentration). Furthermore, from the results of this study, it is evident that the biologically active components responsible for the modulation of drug pharmacokinetics and absorption are most probably concentrated in the polysaccharide component of the *A. vera* gel material although it cannot be directly correlated with the molecular size of the polysaccharide component [38].

### 4.2. Bile Salts

The ability of bile salts to enhance the oral bioavailability of compounds, especially poorly water-soluble drugs, has been discovered many years ago [136,137,138].

The study by Yu, Zhu, Wang, Peng, Tong, Cao, Qiu and Xu [102] investigated the bioavailability of silybin when mixed with bile salt micelles compared with silybin-*N*-methylglucamine alone after oral administration in dogs. The prepared mixed bile salt micelles showed a mean particle size of 75.9 ± 4.2 nm. Silybin-sodium cholate/phospholipid-mixed micelles revealed a very slow release of the silybin, only 17.5% (*w/w*) over 72 h in phosphate buffer (pH 7.4) and 15.6% (*w/w*) in HCl solution (pH 1.2). In spite of this slow release, the relative bioavailability of silybin in the mixed micelles versus silybin-*N*-methylglucamine in dogs was 252% [102].

An in vivo study in diabetic rats was used to investigate the regional-specific intestinal delivery of insulin by co-administration of sodium glycocholate [139]. Insulin (10 UI/kg) was administered intestinally (duodenum, jejunum and ileum) to rats by surgical technique without (control) and with 5% sodium glycocholate. Insulin absorbed from the gastrointestinal tract was investigated by measuring the hypoglycemic effect in the rats at 45 and 60 min post administration. The hypoglycemic effect (100%) of the positive control was specified at 77.9 mg/100 mL glucose at 45 min and 70.5 mg/100 mL glucose at 60 min. For the duodenum region, the insulin control showed a hypoglycemic effect of 71% at 45 min and 84% at 60 min, while the insulin with sodium glycocholate showed hypoglycemic effects of 90% and 95% at 45 and 60 min post administration, respectively. For the jejunum region, the insulin control showed 34% and 54% hypoglycemic effects, while the insulin with sodium glycocholate showed 39% and 60% hypoglycemic effects at 45 and 60 min post administration, respectively. However, administration within the ileum of rats did not demonstrate a significant decrease in blood glucose concentration profiles compared to control. For the ileum, the insulin control showed 9% and 5%, while insulin with sodium glycocholate showed 5% and 8% at 45 and 60 min post administration, respectively [139].

These effects could be contributed to the increased insulin absorption in the presence of the bile salt, potentially by mechanisms of mucus layer modification as well as tight junction modulation [140,141]. Finally, the effects of sodium glycocholate on the intestinal absorption enhancement of insulin demonstrated site-dependent effects with duodenum being the optimal site for insulin oral delivery [139].

### 4.3. Black Cumin

*Nigella sativa*, commonly known as black cumin/caraway, was previously evaluated as a bioenhancer for amoxicillin [142]. *N. sativa* extracts were prepared by cleaning, milling and sieving seeds, followed by extractions with methanol and hexane for 6 h [142]. Everted rat intestinal sacs were used to study the transfer of amoxicillin (6 mg/mL) in PBS (pH 7.4) with or without methanol (3 mg) and hexane (6 mg) extract of *N. sativa* seeds. The amount of amoxicillin transported across the gut was quantified spectrophotometrically at 273 nm [142]. For in vivo studies, amoxicillin (25 mg/kg) was orally co-administered with *N. sativa* hexane extract (25 mg/kg) to rats. Blood samples were collected at 0, 0.25, 0.5, 0.75, 1, 1.5, 2, 4, 6 and 8 h post-dosing, after which UPLC-MS/MS was used to quantify the amount of amoxicillin in rat plasma [142].

Results from the in vitro study demonstrated that both the methanol and hexane extracts of *N. sativa* significantly increased the permeation of amoxicillin, with the latter showing the greatest increase [142]. Hence, hexane extract was selected for in vivo evaluation. Results from the in vivo study also demonstrated a significant increase in amoxicillin plasma concentration in rats. *N. sativa* extract increased the rate and extent of amoxicillin absorption, increasing the *C*_max_ from 4138.251 ± 156.93 to 5995.045 ± 196.28 ng/mL, while AUC_0__→t_ increased from 8890.40 ± 143.33 to 13483.46 ± 152.45 ng/mL·h. It was suggested that this permeation enhancing effect of *N. sativa* might be attributed to the presence of fatty acids [142]. It has previously been demonstrated that fatty acids are able to enhance permeation of low permeable drugs by increasing the fluidity of the apical and basolateral membranes [143]. A similar in vivo study performed on rabbits yielded opposite results. In that study, the *C*_max_ and AUC_0−∞_ of cyclosporine (30 mg/kg) significantly decreased by 35.5% and 55.9%, respectively, after pretreatment with *Nigella* (200 mg/kg). However, a significant increase in cyclosporine clearance (~2-fold) was observed, thus suggesting that intestinal P-gp and/or CYP3A4 are activated in the presence of *N. sativa* [144].

### 4.4. Capsaicin

*In vitro*, in situ and in vivo evaluations of the effect of capsaicin pre-treatment on fexofenadine showed significantly enhanced intestinal absorption of fexofenadine [145]. Non-everted intestinal sacs of rats were employed in the in vitro study, while an in situ single-pass intestinal perfusion study was conducted on rats where the ileal segment (~8–12 cm) was isolated and cannulated. For the in vivo study, the same pre-treatment was applied for 7 days in rats. Results from the non-everted sac study indicated a significant increase in the intestinal transport and P_app_ of fexofenadine in the presence of capsaicin. It was suggested that P-gp efflux inhibition in intestine of rats was the action mechanism, since the results obtained with capsaicin pre-treatment and verapamil, a standard P-gp inhibitor, were comparable [145]. In situ single-pass intestinal perfusion results demonstrated a significant increase in the absorption rate constant, fraction absorbed, and effective permeability of fexofenadine in rats pre-treated with capsaicin and verapamil in comparison with control group [145]. In vivo results showed a significant increase in the AUC and *C*_max_ of fexofenadine orally administered to rats pretreated with capsaicin. Additionally, the apparent oral clearance of fexofenadine was significantly decreased, while *t*_max_ and *t*_1/2_ were unchanged. Findings from this study thus provide in vivo evidence that capsaicin might increase the bioavailability of fexofenadine via the inhibition of P-gp-mediated drug efflux [145].

### 4.5. Caraway

Caraway is obtained from the dried ripe fruit of the plant *Carum carvi*. In an open-label, cross-over in vivo study, the bioenhancing effect of *C. carvi* (Caraway) extract (100 mg) on the pharmacokinetics of rifampicin, isoniazid and pyrazinamide administered as a fixed-dose combination (FDC) was investigated. The Caraway extract increased both *C*_max_ and AUC as indicators of bioavailability for all three drugs, statistically significantly. The increase in *C*_max_ was 32.22%, 36.01% and 33.22% for rifampicin, isoniazid and pyrazinamide, respectively, while the increase in AUC_0–24 h_ was 32.16%, 29.06% and 27.92% for rifampicin, isoniazid and pyrazinamide, respectively. According to the authors, the improvement in bioavailability caused by Caraway extract may be attributed to an enhancement of mucosal to serosal permeation as well as its influence on P-gp efflux [40]. Caraway was shown to act as an inhibitor of P-gp efflux and the main active components were found to be carvone and limonene [146].

### 4.6. Cylcosporine A

Cyclosporine A is a polypeptide consisting of 11 amino acids and was initially derived from a fungus, *Tolypocladium inflatum Gams* [147]. Cyclosporine A is used amongst other indications as an immunosuppresive agent during organ transplantation, to treat autoimmune diseases and in the treatment of certain viral diseases such as Hepatitis C, to name but a few uses. Cyclosporine A has been shown to act as an inhibitor of the efflux transporter, P-gp and as a result may increase the bioavailability of drugs that are substrates for this active efflux transporter [51]. Cyclosporine A has been shown to improve the bioavailability of clopidogrel, an antiplatelet drug, in rats. Co-administration of a dose of 10 mg/kg of cyclosporine A and 30 mg/kg of clopidogrel resulted in a 3.48-fold and 2.83-fold increase in the AUC and *C*_max_ of clopidogrel, respectively. The authors attributed this increase in bioavailability to the inhibition of P-gp-mediated efflux [49].

### 4.7. Chitosan and Derivatives

The oral drug absorption enhancing effects of chitosan and its derivatives have previously been reviewed extensively [148,149]. In brief, several studies have shown that chitosan (including chitosan salts such as chitosan hydrochloride and chitosan glutamate) is an effective oral drug absorption enhancer across in vitro cell models as well as in vivo animal models [42,150,151,152]. The major mechanism of drug absorption enhancement has been shown to involve tight junction modulation to allow for enhanced paracellular uptake of hydrophilic and macromolecular drug compounds. Although a number of studies indicated interactions of chitosan with epithelial cells to open tight junctions [153] through redistribution of F-actin and ZO-1 [148,154], it was shown on HT-29/B6 cells with two-path impedance spectroscopy that the tight junction opening effect of chitosan was due to changes in intracellular pH caused by the activation of a chloride-bicarbonate exchanger [155].

To improve some of the characteristics of chitosan, several derivatives and chemically modified chitosans have been investigated for their drug delivery potential including trimethyl-chitosans, thiolated chitosans, carboxymethyl chitosan and derivatives, hydrophobic chitosans, chitosan succinate and phthalate, PEGylated chitosans and chitosan-enzyme inhibitor conjugates, which have been published in a review previously [156].

### 4.8. Curcumin

Curcumin (diferuloylmethane) is the major curcuminoid contained in the rhizome of *Curcuma longa* L. (turmeric) that has been shown to have several biological and pharmacological activities [157]. A previously published review on pharmacokinetic interactions of curcuminoids with conventional drugs revealed potential interactions via modulation of CYP450 and phase II enzymes as well as P-gp efflux inhibition and potential effects on organic anion transporting polypeptides (OATP). Unfortunately, contrasting results were obtained by different studies ranging from drug absorption enhancement to drug absorption reduction by curcumin [158].

An example of a study where curcumin was found to significantly increase drug bioavailability is an in vivo study in Sprague–Dawley rats that received curcumin (60 mg/kg) for four days prior to drug administration (pre-treatment group), while another group received curcumin (60 mg/kg) once only concomitantly with the drug and the control group received no curcumin. The bioavailability of celiprolol increased statistically significantly in the curcumin pre-treated rats, but not in rats where it was co-administered once only with curcumin. Although the bioavailability of midazolam increased in both the pre-treatment group as well as the co-administered group, it was only statistically significant in the pre-treatment group. Western blot analyses revealed that intestinal P-gp protein expression reduced by 49%, while CYP3A protein content reduced by 42% after 4 days of pre-treatment with curcumin. On the other hand, the hepatic P-gp protein expression increased by 144% and the CYP3A protein content was increased by 91%. Renal P-gp protein levels remained unchanged, but renal CYP3A was enhanced by 41%. Since celiprolol is a P-gp substrate and midazolam is extensively metabolized by CYP3A enzymes, it was deduced from this study that the pharmacokinetics of these two drugs have been changed by curcumin due to down regulation of P-gp and CYP3A in the small intestine [48]. Furthermore, when curcumin was administered at a dose of 10 mg/kg with tamoxifen to rats, the AUC and *C*_max_ values of tamoxifen were significantly increased (i.e., by 64% and 71%, respectively) [159]. Pre-treatment of broiler chickens with 100 mg/kg curcumin for ten days enhanced the absolute and relative bioavailabilities of marbofloxacin in these animals [160]. The P-gp inhibiting effects of curcumin was confirmed in an in vitro study on a human P-gp overexpressing cell line (LLC-GA5-COL300) where the uptake of calcein-AM was increased [161].

### 4.9. Diosmin

Certain flavones (e.g., tangeretin, nobiletin and bergamottin) from citrus fruits have shown P-gp inhibitory effects, which caused pharmacokinetic interactions with drugs that are substrates for this efflux transporter. Diosmin, a flavonoid contained in citrus fruit, was investigated in the Caco-2 cell model for P-gp inhibitory effects using rhodamine 123 and digoxin as model compounds. Rhodomine 123 accumulation in the Caco-2 cells was investigated in the absence and presence of diosmin. At a concentration of 50 µM, diosmin caused a 494 ± 8.4% increase in the cellular accumulation of rhodamine 123 in reference to the control. To further investigate the initial observation of P-gp-mediated efflux inhibition by diosmin, bi-directional transport (i.e., in the apical-to-basolateral [A-B] and basolateral-to-apical [B-A] direction) of digoxin across Caco-2 cell monolayers was conducted in the absence and presence of diosmin. At a concentration of 50 µM diosmin, the P_app (A–B)_ was significantly increased from 2.8 ± 0.07 × 10^−6^ cm/s to 9.5 ± 0.06 × 10^−6^ cm/s, while the P_app (B–A)_ was significantly decreased from 41.1 ± 1.20 × 10^−6^ cm/s to 17.1 ± 0.10 × 10^−6^ cm/s. These P_app_ values corresponded to transport ratio values of 15.2 in the absence and 2.3 in the presence of diosmin. Based on the results of this study, it is clear that diosmin act as a P-gp efflux transport inhibitor and as such has the potential to improve the bioavailability of drugs that are substrates for P-gp [50].

In a more recent study, the effect of diosmin on the in vivo pharmacokinetics of fexofenadine in rats was investigated. In this study, rats were pre-treated for 7 days with diosmin (50 mg/kg) where after fexofenadine (10 mg/kg) was administered on day 8. Pre-treatment with diosmin resulted in a 2.5- and 2.2-fold increase in *C*_max_ and AUC_0-__∞_, respectively. The enhanced bioavailability of fexofenadine in this study was attributed to the fact that diosmin is a potent P-gp inhibitor [162].

### 4.10. Emodin

Anthraquinones such as emodin (1,3,8-trihydroxy-6-methyl-anthraquinone) occur naturally in a wide variety of plants and herbs such as *Rheum* *palmatum* [163], *Polygonum* *multiflorum* [164], *Polygonum cuspidatum* [165], *Cassia obtusifolia* [166] and *Aloe vera* [167]. Administration of emodin may lead to synergistic and/or inhibitory effects on P-gp-related efflux and/or modulation of multidrug-resistance associated protein 1, 2 and 3 (MRP1, MRP2 and MRP3) and these effects may have a profound effect on the bioavailability of co-administered substrate molecules [163,168,169,170,171].

In an extensive study conducted by Min, et al. [172], potential interactions between emodin and rhodamine 123 were investigated in various in vitro models. In this study, human myelogenous myeloid leukemia cells (K562), the P-gp over-expressing adriamycin resistant K562 cells (K562/ADM) and Caco-2 cells were used as in vitro models to investigate if the co-administration of emodin at various concentrations had any significant effect on the intracellular accumulation of rhodamine 123 (1 μM). The results showed that addition of emodin increased the intracellular accumulation of rhodamine 123 in a concentration-dependent manner in both the Caco-2 and K562/ADM cell models. The extent of the accumulation of rhodamine 123 in Caco-2 cells by emodin (20 μM) was comparable to that of a much higher verapamil concentration (200 μM). Binding site competition between emodin and rhodamine 123 on P-gp was also investigated. The results showed that the calculated *K_i_* values increased consistently in conjunction with increasing rhodamine 123 concentrations (0 to 20 μM), which indicated that a competitive interaction occurred between emodin and rhodamine 123. It was concluded that emodin and rhodamine 123 shared the same R-binding site on P-gp [172].

An MTT method was also used in conjunction with the K562/ADM cell line to investigate if the addition of emodin could potentially reverse P-gp-mediated MDR. The results showed that the addition of emodin (20 μM), alone or in combination with 10 μM adriamycin, exerted down regulation of P-gp expression in comparison to the control group. The authors concluded that emodin could potentially reverse P-gp-mediated MDR due to inhibition of P-gp expression in K562/ADM cells and also by competitive interactions between emodin and rhodamine 123 at the R-binding site of P-gp. The results confirmed that emodin is an effective inhibitor of P-gp and its effects are mediated either by direct binding to P-gp and subsequent weakening of the P-gp-mediated efflux function or by indirect mechanisms related to a reduction in P-gp expression [172].

In another study, it was also shown that the addition of emodin to the Caco-2 cell model had the ability to decrease P-gp-mediated efflux, but additionally it was also reported that P-gp expression was mediated via the MAPK/AP-1 pathway by means of COX-2 inhibition [173]. An in vitro transport study across rat intestinal tissues using an Ussing-chamber technique showed that the transport of digoxin was decreased in the presence of emodin while the transport of teniposide was increased in the presence of emodin. The authors concluded that emodin was an inducer of P-gp-related efflux and also an inhibitor of MRP3 [174].

### 4.11. Gallic Acid Ester

Gallic acid is a water soluble phenolic acid which is found in grapes and in the leaves of various plants and forms part of a larger group of plant polyphenols known as gallotannins [175]. Gallotannins are transformed in the gastrointestinal tract by hydrolysis to form free gallic acid [176]. Various health benefits are associated with the intake of gallic acid and it is reported in the literature to exert anti-cancer, anti-inflammatory, cardio-protective and anti-diabetic properties [175]. Gallic acid has been reported to exhibit inhibitory effects on P-gp-mediated drug efflux and also on CYP450-related isozymes such as CYP3A4 [177,178,179].

In vitro and in situ transport studies were conducted to investigate the effects of gallic acid on the transport of diltiazem. The in vitro studies entailed the use of non-everted gut sacs from Wistar rat intestinal tissue. The results from the in vitro study showed that the apparent permeability of diltiazem had increased in the presence of gallic acid by 4.4-, 5.1- and 4.9-fold in the respective segments of the duodenum, jejunum and ileum of non-everted intestinal gut sacs when compared with the control group [180].

For the in situ single-pass intestinal perfusion study, rats were randomly divided into four groups (*n* = 5) (control, standard inhibitor, and pre-treated with either gallic acid or ellagic acid). Perfusion of the cannulated intestinal segment was performed using phosphate buffered saline (pH = 7.2) containing diltiazem (100 μg/mL) and phenol red (50 mg/mL). A constant flow rate of 0.2 mL/min was maintained for a period of 90 min and samples were collected at 10 min intervals. The results from the in situ study showed that pre-treatment with gallic acid (50 mg/kg) for 7 days resulted in a significant (*p* < 0.05) increase in diltiazem transport in rats when compared to the control group (diltiazem alone) following pre-treatments with gallic acid for 7 days. The *C*_max_, AUC_0–t_, and AUC_0–__∞_ of diltiazem were increased by 1.90-, 2.06- and 2.08-fold, respectively (Athukuri and Neerati., 2018). Based on these results, it was suggested that gallic acid was most likely a dual inhibitor of both P-gp and CYP3A4 and that this dualistic effect may have resulted in the significant enhancement in the bioavailability of diltiazem [180].

The transport of metoprolol was assessed in a similar in situ single-pass intestinal perfusion study to evaluate the pharmacokinetic parameters of orally administered metoprolol in Wistar rats. The rats were pre-treated with gallic acid (50 mg/kg) for 7 days before the start of the study. A significant increase in the *C*_max_ and AUC values of metoprolol were evident. It was concluded that gallic acid had enhanced the oral bioavailability by inhibiting CYP2D6 activity in the liver, which caused reduced metabolism of metoprolol [181].

### 4.12. Genistein

The flavonoid genistein (5,7-Dihydroxy-3-(4-hydroxyphenyl)chromen-4-one) is a plant-derived (*Glycine max* and *Pueraria lobata*) isoflavone and a phytoestrogen, frequently ingested by humans [54,55,182]. Genistein has very poor bioavailability in itself and although some studies have suggested that genistein may have anticancer effects, these effects could not be obtained in clinical studies because of its low bioavailability [183]. Genistein is purported to be an enhancer of bioavailability of drugs, since it has been shown to inhibit efflux of P-gp, BCRP and MRP2 transporters. These properties were investigated in a Caco-2 antiparasitic agent transport study, where taxol (a P-gp substrate) was applied in combination with 33 or 100 μM genistein [184]. Although no inhibition of transport could be measured for 33 μM genistein, 100 μM genistein resulted in 20% inhibition. It was subsequently determined that although genistein inhibited P-gp, it did not appear to be a substrate of P-gp. Another study showed 10–30 µM genistein increased sensitivity of a P-gp hyper-expressing drug resistant human cervical carcinoma cell line (KB-V1) to vinblastine and paclitaxel, while reducing efflux of Rhodamine 123 and vinblastine in a dose-dependent manner (10–200 µM) [185]. However, no correlating change in P-gp expression was observed following exposure to genistein, indicating only P-gp activity was modulated. Li and Choi [55] later demonstrated that a single oral dose of 30 mg/kg paclitaxel 30 min after ingesting 3.3 mg/kg or 10 mg/kg genistein, significantly increased the AUC by 54.7% in male Sprague–Dawley rats. This was as a result of decreased plasma clearance (35.2%), thereby increasing systemic exposure. The same effect was seen when the paclitaxel was administered intravenously (3.3 mg/kg). It was proposed that the genistein inhibited the efflux transporters and metabolic enzymes for which paclitaxel is known to be a substrate, probably being P-gp and CYP3A [55,182].

Genistein also inhibited efflux of 2′,7′-bis-(carboxypropyl)-5(6)-carboxyfluorescein (BCPCF), an MRP1 substrate, from human erythrocytes. Genistein displayed an IC_50_ concentration of 50–70 μM for BCPCF efflux. It was reported that genistein inhibited MRP1 efflux transporters, but that this modulation was substrate sensitive [186].

Genistein has been shown to inhibit the biotransformation and intestinal efflux of (-)-epigallocatechin-3-gallate (EGCG) in more recent studies. EGCG is a flavonoid in various foods and beverages, but especially in *Camellia sinensis* (green tea), and has been shown to have several therapeutic effects such as anti-cancer, anti-viral and anti-inflammatory effects, but it has very poor oral systemic absorption [187]. Lambert, Kwon, Ju, Bose, Lee, Hong, Hao and Yang [54] demonstrated an increase in cytosolic EGCG of 2- to 5-fold in the presence of genistein (20 μM), in HT-29 human colon cancer cells. They also treated CF-1 mice with 200 mg/kg genistein and 75 mg/kg EGCG, resulting in increased plasma half-life and maximal concentration of the EGCG. However, in a male adenomatous polyposis coli (APC) min/+ mouse model, this combination also enhanced tumor genesis.

An in vitro and in vivo study treated Mardin-Darby canine kidney (MDCK-II) cells and Assaf sheep with the antibacterial fluoroquinolone, danofloxacin, concomitantly with genistein [188]. Danofloxasin is exclusive for animal use and is actively secreted into milk. The in vitro transport study showed that genistein inhibited BCRP transport of danofloxasin efficiently, while the in vivo study indicated no change in danofloxasin plasma levels following isoflavone supplementation. A prolonged diet of soy, however, did reduce antimicrobial concentrations in the milk.

Important to note are the findings of Chen, et al. [189], which indicated that genistein can induce the phase II drug-metabolizing enzyme, sulfotransferase. Enzymatic activity, protein levels and mRNA expression were evaluated in a transformed human liver cell line (HepG2) and a colon carcinoma cell line (Caco-2). It was concluded that gene expression (SULT1A1 and SULT2A1) was induced by genistein in a time and dose-dependent (0–25 μM) manner. Contrary, approximately 0.1 µM genistein inhibited human liver phenol sulfotransferase by 50% in a competitive manner [190].

### 4.13. Gokhru Extract

Gokhru extract, a popular plant extract used in Ayurvedic medicines, is derived from *Tribulus terrestris* Linn (Zygophyllaceae) [56,191]. Some of the phytochemicals previously identified in *T. terrestris* include saponins, steroids, flavonoids and carboline alkaloids. Gokhru extract is traditionally used as a diuretic, anti-inflammatory, anabolic, spasmolytic, muscle relaxant, hypotensive, and hypoglycemic agent, but it has been reported to influence bioavailability of co-administered drugs.

One study investigated the effect of Gokhru extract on the absorption of metformin hydrochloride (HCl) in an everted sac model [56]. Metformin is an anti-diabetic drug that is known to be highly soluble, but poorly membrane permeable (BCS class III). In this study, the extract was prepared from dried plant material (fresh fruits, leaves and stems) with hot ethanol. The results showed increased absorption of metformin in the presence of the Gokhru extract, and the authors suggested the major saponin component in the extract may have contributed largely to this increase in drug transport. Saponins consist of one or more sugar chains, connected to a steroid or triterpenoid aglycon. It can solubilize cholesterol, while maintaining most of the structure of the cell membranes, thereby permeabilizing it to increase membrane permeability [57].

A similar study formulated metformin HCl tablets (175–500 mg) with varying concentrations Gokhru extract (0–100 mg), which were then investigated using a chicken intestine everted sac model. In this study, the drug absorption enhancement properties of Gokhru extract was confirmed with increased metformin permeation across the chicken intestinal membranes from 29% to 54% [57].

A methanol extract was prepared from dried *T. terrestris* leaves and applied together with salicylic acid (aspirin) to the mucosal side of the goat intestinal tissues used in an everted sac technique. The Gokhru extract increased aspirin transport and it was proposed that the permeability enhancing action was a result of the saponins effects on the membranes [191].

### 4.14. Grapefruit Juice

The pharmacokinetic interaction between grapefruit (*Citrus paradisi*) juice and the calcium channel antagonist, felodipine, was discovered serendipitously during an in vivo clinical study in 1989 that was designed to evaluate the potential interaction between ethanol and felodipine. Grapefruit juice was used to mask the taste of ethanol in this study. Concomitant intake of felodipine with grapefruit juice caused higher anti-hypertensive effects as well as higher felodipine plasma concentrations. This observation led to a follow-up pilot study in a single volunteer where felodipine plasma concentrations were found to be more than 5-fold higher when it was taken with grapefruit juice than with water [192]. Since this initial discovery, more than 85 drugs have shown to interact with grapefruit juice in terms of pharmacokinetics of which most experienced increased plasma concentrations [193].

In a study on borderline hypertensive patients where the effect of different fruit juices was evaluated on felodipine pharmacokinetics, it was shown that grapefruit juice, but not orange juice, could markedly increase felodipine’s bioavailability parameters (both *C*_max_ and AUC were increased). In addition, grapefruit juice reduced the ratio of dehydrofelodipin (the primary metabolite of felodipine produced by CYP3A4) to felodipine. This effect was not observed for intravenous administration of felodipine with grapefruit juice, which indicated selective inhibition of pre-systemic metabolism involving CYP3A4 [194].

Several studies showed that three groups could be distinguished with respect to the effect of grapefruit juice on the pharmacokinetic parameters of drugs, namely “increase”, “decrease” and “no change” [195]. The uptake and bioavailability enhancement effect of grapefruit juice was shown in a study where both in vitro and in vivo models were used. Lyophilized freshly-prepared grapefruit juice significantly increased the uptake of doxorubicin into human uterine sarcoma (MES-SA/DX5) cells and significantly increased the bioavailability of timolol maleate in rabbits [196]. However, it was shown that both regular strength and double strength grapefruit juice did not have a significant effect on the pharmacokinetic parameters of simvastatin when administered concomitantly to Sprague–Dawley rats in a dose of 20 mg/kg over 28 days. On the other hand, simvastatin plasma concentrations were elevated by double strength grapefruit juice when the drug was administered at a dose of 80 mg/kg [197], indicating a dose-dependent pharmacokinetic interaction. In another in vivo study, grapefruit juice decreased the oral bioavailability of fexofenadine [58].

Results from different studies revealed, over time, that the pharmacokinetic effect of several forms of grapefruit (i.e., whole fruit, fresh fruit juice or frozen concentrate) is drug-specific and it may in some cases be concentration-dependent. It was also shown that the lower the inherent bioavailability of the drug due to pre-systemic metabolism, the higher the chance is that the interaction with grapefruit can be dangerous [193].

One of the main mechanisms of action of grapefruit juice by which the bioavailability of drugs (e.g., felodipine) is increased, is by mechanism-based inhibition of CYP3A4 in the enterocytes of the small intestine and hepatocytes of the liver [58,198]. It was also shown that grapefruit juice could inhibit the P-gp efflux transporter and thereby increase the bioavailability of drugs that are substrates for this efflux transporter (e.g., talinolol) [199,200]. Furthermore, a study on 10 volunteers that received grapefruit juice three times a day for 6 days revealed that their small intestinal CYP3A4 protein expression reduced by 62%, but not their liver CYP3A4 protein expression. This selective down regulation of CYP3A4 correlated well with the *C*_max_ increase of felodipine after grapefruit juice consumption relative to that taken with water [201]. On the other hand, it was found that grapefruit juice has the ability to inhibit uptake transporters such as OATP, specifically OATP1A2 and OATP2B1, and thereby it can decrease the absorption of certain drugs (e.g., fexofenadine) [202,203].

### 4.15. Lycopene

The effect of a lycopene-containing nano-formulation containing simvastatin on low density lipoprotein (LDL) was investigated in mildly hypercholesterolemic patients [60]. The aim of this study was to evaluate lycopene as a vector of intrahepatic transport to specifically enhance the hepatic bioavailability of simvastatin. This is important, since hepatic cholesterologenesis is the main site of statin action. Furthermore, the plasma lipid profile is not affected by extrahepatic inhibition of HMG-CoA reductase, and extrahepatic toxicity is observed with increasing statin concentrations [60]. A total of 10 patients with moderately increased plasma LDL levels (150 to 200 mg/dL) received 20 mg of either unmodified simvastatin or lycosome-formulated statin (Lyco–Simvastatin) daily. Plasma samples were obtained after 30-day treatment and analyzed for lipids. The results demonstrated that the solubilized lycosome nanoparticles increased the intestinal absorption rate in comparison with unmodified simvastatin and were able to bind hepatocyte membranes. The lycopene-containing simvastatin formulation significantly (*p* = 0.0049) reduced LDL levels in the hypercholesterolemic patients as compared to unmodified simvastatin [60].

The mechanism of action of lycopene is enhanced hepatic uptake via a dual carotenoid/LDL receptor-mediated mechanism [60]. It was shown that lycopene crystals and/or lycopene-containing nanoparticles (lycosomes) became incorporated into chylomicrons upon absorption. The chylomicrons are then distributed by lymph and likely undergo a dual receptor-mediated uptake associated with the lycopene core. The latter is a powerful ligand for carotenoid receptors that are expressed by hepatocytes and therefore promoted intrahepatic delivery of lycosome-formulated statins [60]. Enhanced hepatic uptake of lycostatin is also possible due to a second pathway of intrahepatic uptake involving an LDL-receptor mechanism. Chylomicrons and their enzymatically degraded products, LDL and very low density lipoprotein (VLDL), contain ApoB that mediates their transport inside hepatocytes using the LDL receptor [60]. Although further research related to the pharmacology of Lyco-Simvastatin is needed, lycopene seems to a promising bioenhancer for targeted hepatic delivery of simvastatin [60].

### 4.16. Lysergol

Lysergol (9,10-Didehydro-6-methylergoline-8-*O*-methanol) is a compound present in several higher plants (e.g., *Ipomoea violacea*, *I. muricata* and *Rivea corymbosa*) and lower fungi (e.g., *Claviceps*, *Penicillium* and *Rhizopus*) [182,204]. Lysergol is traditionally used as a psychotropic, analgesic, analeptic, hypotensive and immuno-stimulant. It usually maintains normal blood flow through its vaso-activity and can promote drug absorption from the gastrointestinal tract [205]. An in vivo study by Shukla, Malik, Jaiswal, Sharma, Tanpula, Goyani and Lal [62] indicated that lysergol increased the bioavailability and decreased the clearance of curcumin significantly. This was followed by in vitro mechanistic studies using rat liver microsomes and probe substrates for P-gp and BCRP transporter proteins. The results suggested that BCRP, rather than P-gp, was inhibited by lysergol. In situ single-pass intestinal perfusion studies of the P-gp substrate, digoxin, and the BCRP substrate, sulfasalazine, were then performed in Sprague–Dawley rats following pre-treatment with 20 mg/kg lysergol. These studies supported the conclusion that P-gp was not involved in the permeation-enhancing effect of lysergol, but that BCRP was inhibited [62].

Lysergol (2–10 μg/mL) isolated from *I. muricata* seeds with methyl alcohol has been shown to act as a bioenhancer of antibiotics. The authors showed that rifampicin and tetracycline intestinal transport could be enhanced (2.96–8.53 fold) by lysergol in vitro [204]. Lysergol also increased the oral bioavailability of the quaternary protoberberine alkaloid, berberine, in male Sprague–Dawley rats. Berberine is used for numerous ailments, including bacterial infection, intestinal parasitic infection and diarrhea, but it is very poorly absorbed in the intestines and extensively metabolized. The study indicated that 20 mg/kg lysergol could increase the bioavailability of the berberine [61].

### 4.17. Naringin and Bergamottin

Certain phytochemicals such as naringin (a flavonoid) and bergamottin (a furanocoumarin) contained in grapefruit have been associated with the pharmacokinetic interactions observed for grapefruit juice co-administered with certain drugs [195]. When paclitaxel has been orally co-administered with naringin to Sprague–Dawley rats, the paclitaxel plasma concentrations increased statistically significantly. Since naringin affected the bioavailability of paclitaxel similar to that of quercetin, a known inhibitor of CYP3A and P-gp, it was deduced that naringin enhanced the bioavailability of paclitaxel by means of inhibition of CYP3A metabolism and P-gp efflux transporters [66]. Similarly, the pharmacokinetic parameters of diltiazem (AUC and *C*_max_) were increased by 2-fold when the rats were pre-treated with naringin (administered 30 min prior to diltiazem) compared to the control group. In addition, the AUC metabolite-to-parent ratio decreased by 30% in the presence of naringin compared to the control. This confirmed the ability of naringin to inhibit metabolism of diltiazem [64].

The CYP3A4 inhibition activities of bergamottin and 6′,7′-dihydroxybergamottin were investigated in a human intestinal microsome study on two model compounds namely testosterone and midazolam. Bergamottin was identified as a substrate-dependent reversible inhibitor, but a substrate-independent mechanism-based inhibitor of CYP3A4. On the other hand, 6′,7′-dihydroxybergamottin was found to be a substrate-independent reversible and mechanism-based CYP3A4 inhibitor [206].

### 4.18. Palmitoyl Carnitine Chloride

Initial drug absorption enhancement studies on palmitoyl carnitine, a fatty acid ester of carnitine, showed that it is capable of intestinal drug absorption enhancement by means of tight junction opening (paracellular transport) and disruption of brush-border membrane lipids (transcellular transport) [207,208]. An in depth study on Caco-2 cell monolayers indicated that lytic effects and reduction in cell viability accompanied transport enhancement of hydrophilic macromolecules at all concentrations of palmitoyl carnitine that were investigated. It was concluded that the alteration of the tight junctional network, which was shown by TEER reduction and confocal laser scanning microscopic localization of ZO-1, occurred secondary to the interaction of palmitoyl carnitine with the membrane. Since complete recovery of TEER could not be obtained, the use of palmitoyl carnitine for drug absorption enhancement should be considered with caution [209].

### 4.19. Piperine

Piperine is an alkaloid contained in black pepper (*Piper nigrum*) and long pepper (*Piper longum*) [11]. Piperine can probably be considered as one of the world’s first bioenhancers since its use dates as far back as the 7th century BC [205,210]. Piperine is traditionally used for its anti-inflammatory, anti-pyretic, anti-fungal, anti-diarrheal and anti-cancer effects [211]. Possible mechanisms by which piperine caused bioenhancing effects include alteration of membrane dynamics, inhibition of P-gp efflux, and inhibition of gastrointestinal and hepatic metabolism [11,83,210].

A study that investigated the effects of piperine on the serum levels of resveratrol (3,5,4′-trihydroxystilbene) when orally co-administered to C57BL mice, showed that AUC and *C*_max_ were increased by 229% and 1,544%, respectively [77]. Jin and Han [76] investigated the bioavailability changing effects of orally administered piperine in rats when co-administering fexofenadine either orally (10 mg/kg) or intravenously (5 mg/kg). The results suggested that there were no significant variations in the *C*_max_ of fexofenadine or its half-life, but the oral exposure (AUC) of fexofenadine was almost doubled. It was deduced from this study that the effects of piperine are more prominent after oral administration (likely due to the P-gp-mediated efflux inhibition and metabolism inhibition in the gastrointestinal epithelium) than after intravenous administration (due to hepatic metabolism inhibition) [76].

### 4.20. Quercetin

Quercetin, which is a CYP3A4 and P-gp inhibitor, has shown the ability to significantly enhance the bioavailability of orally administered tamoxifen and 4-hydroxytamoxifen in rats [95]. In that study, tamoxifen (10 mg/kg) was orally administered with or without quercetin (2.5, 7.5 and 15 mg/kg) in rats. Tamoxifen (10 mg/kg) was added to 1.5 mL distilled water for the control solution, whereas the required quercetin dose was dissolved in 1 mL distilled water to prepare quercetin suspensions for oral administration. Plasma concentrations of tamoxifen were measured in blood samples (0.5 mL) collected at 0, 0.5, 1, 2, 3, 4, 5, 6, 8, 12, 24 and 36 h after tamoxifen administration using HPLC [95].

The relative bioavailability of tamoxifen was 1.35- and 1.61-fold higher, with an absolute bioavailability of 20.2% and 24.1% with 2.5 and 7.5 mg/kg quercetin, respectively. These changes in bioavailability were significant (*p* < 0.05). Interestingly, higher concentrations of quercetin (15 mg/kg) did not induce any significant changes. No significant changes in the terminal half-life (*t*_1/2_) and the time to reach the peak concentration (*T*_max_) of tamoxifen was observed when co--administered with quercetin. When 4-hydroxytamoxifen, one of tamoxifen’s metabolites, were co-administered with quercetin (7.5 mg/kg), a significant increase in the AUC of 4-hydroxytamoxifen was observed. These results suggested that MDR efflux and first-pass metabolism of tamoxifen was inhibited by quercetin [95].

Another in vivo study demonstrated an inhibitory effect on P-gp efflux of fexofenadine when it was co-administered with quercetin [91]. Twelve healthy subjects received an oral dose of quercetin (500 mg) or placebo 3 times daily for 7 days. On the 7th day, a single dose of 60 mg fexofenadine was administered orally with 240 mL water under fasting conditions of more than 10 h. The healthy volunteers received the oral administration of fexofenadine while in the sitting position, and remained seated for 4 h [91]. Four and 10 h after dosing, subjects received standardized meals with only minor amounts of flavonoids, so as not to influence the absorption of fexofenadine or quercetin. Subsequently, blood samples were drawn immediately before, as well as 0.25, 0.5, 1, 1.5, 2, 2.5, 3, 4, 6, 8, 12, and 24 h after fexofenadine administration. Urine samples were collected during time intervals of 0–2, 2–4, 4–8, 8–12, and 12–24 h after dosing. Plasma and urinary fexofenadine concentrations were quantified using HPLC [91].

Results indicated that quercetin significantly enhanced the mean plasma concentrations of fexofenadine. The AUC of plasma fexofenadine increased by 55% from 2,005.3 to 3,098.6 ng.h/nL (*p* < 0.001) in the presence of quercetin. The *C*_max_ was similarly increased by 68% from 295.3 to 480.3 ng/mL (*p* = 0.006) when co-administered with quercetin. After quercetin treatment, the oral clearance of fexofenadine was significantly decreased by 37% from 61.4 to 38.7 L/h (*p* < 0.001). However, no differences in the renal clearance and half-life were observed between the groups receiving placebo versus quercetin [91].

In summary, quercetin can be used as a bioenhancer for enhanced intestinal absorption, and hence bioavailability, of tamoxifen [95] and fexofenadine [91]. The action mechanisms employed by quercetin are MDR transporter efflux inhibition, as well as first-pass metabolism inhibition [95]. If the results obtained with tamoxifen from the rats’ model is confirmed in the clinical trials, the dose of tamoxifen should be adjusted for potential drug interactions when quercetin or the quercetin-containing dietary supplements are simultaneously consumed with this drug [95]. Although the inhibitory effect of quercetin on P-gp-mediated efflux of fexofenadine was demonstrated in the short-term (7 days), it is uncertain whether long-term use of quercetin will yield the same results [91].

### 4.21. Quinidine

A research study using everted gut sacs demonstrated that quinidine could significantly enhance the absorption of paeoniflorin (20 µM) [97]. Increased absorption of paeoniflorin was observed with an increase of incubation time, indicating that the absorption of this compound was time-dependent. In addition, the absorptive profiles with different concentrations of paeoniflorin indicated that absorption increased with increasing concentrations, but saturation of the in vitro gut sac system was observed at a concentration of ~80 µM. This saturation indicates that transport of paeoniflorin might be facilitated by an active transporter or carrier [97]. Co-administration of quinidine (1.3 mM) and paeoniflorin (20 µM) yielded a 1.5-fold increase in the absorption of paeoniflorin after 45 min of incubation. Absorption of paeoniflorin in the intestine could thus be significantly enhanced by co-administration of quinidine [97].

### 4.22. Resveratrol

A recent study demonstrated that resveratrol could significantly increase rat intestinal absorption of methotrexate in vivo and in vitro [99]. Furthermore, resveratrol was also able to inhibit efflux transport and decrease renal clearance of methotrexate. The bidirectional transport of methotrexate across mock-MDCK, MDR1-MDCK, and MRP2-MDCK cell monolayers were evaluated using an experimental buffer solution containing methotrexate (10 µM) and/or resveratrol (10 µM). This experimental buffer solution was added to the donor chamber, either 400 µL apical or 600 µL basolateral chamber, respectively. At time intervals of 0.5, 1, 2, and 3 h, 50 µL samples were removed from the opposite (receiver) chamber and replenished with 50 µL fresh Hanks’ balanced salt solution (HBSS, pH 7.4) [99].

Additionally, the in vitro uptake of methotrexate in the absence and presence of resveratrol in kidney slices and Caco-2 cells were determined by liquid chromatography-tandem mass spectrometry (LC-MS/MS). To clarify the action mechanism of resveratrol, transporter uptake assays were similarly performed using mock-human embryonic kidney (HEK) 293 cells, human organic anion transport 1 (hOAT1)-HEK293 cells and hOAT3-HEK293 cells. The transport buffer contained 10 μM methotrexate, *p*-amino hippuric acid (PAH), Penicillin G (PCG) or resveratrol. PAH and PCG are specific substrates of OAT1 and OAT3, respectively [99].

The in vivo absorption experiment in rats entailed an oral administration of 5 mg/kg methotrexate dissolved in 2% sodium bicarbonate with or without resveratrol (100 mg/kg, dissolved in 0.5% sodium carboxymethyl cellulose) and brought up to volume with normal saline. Blood samples were collected at 5, 10, 20, 30, 60, 120, 240, 360, 480, and 600 min to determine plasma concentrations of methotrexate. In order to establish the renal excretion of methotrexate, a solution of methotrexate (5 mg/kg) and/or resveratrol (10 mg/kg) dissolved in normal saline solution of hydroxypropyl-β-cyclodextrin was intravenously administered to rats. Again, blood samples were collected at the specified times to determine the plasma concentration of methotrexate. Furthermore, urine was collected at 2, 4, 6, 8, 10, 12, 16, and 24 h after dosing to determine the concentration of methotrexate excreted through urine [99].

In vivo results indicated that a significant increase in the plasma exposure of methotrexate was observed when methotrexate was orally or intravenously co-administered. In vitro results using the rat everted gut sac model demonstrated that serosal concentrations of methotrexate increased significantly in the presence of resveratrol, as well as verapamil and/ or CDF, inhibitors of P-gp and MRP2, respectively. These results thus suggest that resveratrol can enhance methotrexate intestinal absorption by inhibiting P-gp or MRP2. Furthermore, co-administration of methotrexate and resveratrol resulted in a 37.3% decreased cumulative urinary excretion of methotrexate, indicating that resveratrol could inhibit renal excretion of methotrexate. To clarify the action mechanism of the latter, methotrexate uptake experiments were conducted using rat kidney slices. Results from this experiment showed significantly decreased uptake of methotrexate in the presence of resveratrol and probenecid, a well-known inhibitor of OATs. Furthermore, PAH and PCG (substrates of OAT1 and OAT3, respectively), as well as methotrexate uptake was significantly inhibited by resveratrol in hOAT1-HEK293 or hOAT3-HEK293 cells. These results indicated that resveratrol was able to inhibit OAT1/3, which were target transporters involved in drug-drug interactions between methotrexate and resveratrol in the kidney. The rate of basal-to-apical transepithelial methotrexate transport in MDR1-MDCK and MRP2-MDCK cells increased 32.5- and 20.8-fold, respectively, in the presence of resveratrol. These results thus indicated that P-gp and MRP2 are also target transporters of methotrexate, which is inhibited by resveratrol [99].

In vivo and in vitro results indicated that resveratrol could significantly increase the absorption of methotrexate in the intestine, and also decreased methotrexate renal clearance. Uptake, transport and renal clearance studies confirmed that the mechanisms of action of resveratrol entailed inhibition of P-gp, MRP2, OAT1 and OAT3. This corresponds with previous studies demonstrating that methotrexate is mainly eliminated rapidly by OAT1 and OAT3 in the unchanged form into urine [212,213].

A recent study demonstrated contradictory results where resveratrol stimulated P-gp efflux of saquinavir in MDR1-expressing Madin–Darby canine kidney (MDCKII-MDR1) cells [214]. In this study, MDCKII-MDR1 cells were incubated with 50 μM saquinavir and/or resveratrol (1, 10, 33, and 100 μM) or verapamil (50 μM) at 37 °C for 4 h. Verapamil, a P-gp inhibitor, served as the positive control. Additionally, an in vitro metabolism experiment in microsomes assessed the effect of resveratrol on the intestinal CYP3A-mediated metabolism of saquinavir. In order to assess the effects of resveratrol on the pharmacokinetic profiles of saquinavir, 30 mg/kg saquinavir was orally administered, with or without resveratrol (20 mg/kg), to rats. Saquinavir was suspended in solvent (20% ethanol, 30% propylene glycol, and 50% saline) at a concentration of 6 mg/mL, whereas resveratrol was suspended in saline with 30% polyethylene glycol 400 at 20 mg/mL concentration. Subsequently, blood samples were collected at 0, 0.25, 0.5, 1, 2, 4, 8, 12, and 24 h after drug administration, and analyzed with LC-MS/MS [214].

Results demonstrated significantly decreased saquinavir intracellular concentrations in the presence of resveratrol in a concentration-dependent manner, which indicated that resveratrol stimulated P-gp-mediated efflux of saquinavir. Results from the metabolism experiment demonstrated a significant increase in residual saquinavir in the presence of resveratrol in a dose-dependent manner. This suggests that resveratrol has a concentration-dependent inhibitory effect on the intestinal CYP3A-mediated metabolism of saquinavir. Oral co-administration of resveratrol (20 mg/kg) decreased the mean AUC_0–∞_ of saquinavir by ~31%, while the mean apparent systemic clearance (CL/F) was increased by ~51%. However, both these changes were not statistically significant (*p* > 0.05) [214].

### 4.23. Sinomenine

Previous research using everted gut sacs demonstrated that sinomenine at 16 and 136 µM concentrations could significantly enhance the absorption of paeoniflorin (20 µM) by 1.5- and 2.5-fold, respectively [97]. Similarly, the absorption of digoxin (13 µM) could significantly be enhanced by 2.5-fold in the presence of sinomenine (136 µM). Increased absorption of paeoniflorin was observed with increase of incubation time, indicating that the absorption of this compound was time-dependent. In contrast, absorptive profiles with different concentrations of paeoniflorin indicated that absorption increased with increasing concentrations, but saturation of the in vitro gut sac system was observed at a concentration of ~80 µM [97]. This saturation indicated that transport of paeoniflorin might be facilitated by an energy-dependent carrier. Enhanced bioavailability of paeoniflorin (150 mg/kg) in the presence of sinomenine (90 mg/kg) has also previously been demonstrated in vivo in rats, yielding a 12-fold increase [100].

In order to investigate the potential mechanism underlying the observed enhanced absorption of paeoniflorin, two Pg-inhibitors (verapamil or quinidine) and a P-gp substrate (digoxin) was employed. Co-administration of verapamil (20 µM) and paeoniflorin (20 µM) yielded a 2.1-fold increase in the absorption of paeniflorin after 45 min of incubation. Similarly, when quinidine (1.3 mM) was co-administered, a 1.5-fold increase in the absorption of paeoniflorin was observed. Transportation and absorption of paeoniflorin in the intestine could thus be significantly enhanced by inhibition of P-gp by verapamil and quinidine [97]. Sinomenine (136 µM) was also able to significantly enhance the absorption of 13 µM digoxin, which is a well-known P-gp substrate-like drug, by 2.5-fold after 45 min of incubation.

### 4.24. Sodium Caprate (Fatty Acid)

Enhancement of sodium caprate on the intestinal absorption of berberine has been studied by in situ, in vitro and in vivo experiments [101]. Results from the recirculating perfusion model demonstrated an increased absorption rate of berberine (50 µmol/L) at 4 h from 9.3% to 18.5% in the presence of sodium caprate. In comparison, 100 µmol/L and 200 µmol/L berberine with sodium caprate (0.2% *w/v*) showed increased absorption rates of 13.1% and 20.1%, respectively. The in vitro everted rat gut sac experimental results showed that small amounts of berberine is absorbed in the intestine, with the highest and lowest amounts being absorbed in the jejunum and ileum, respectively. In the presence of sodium caprate, absorption of berberine was rapidly and significantly increased after 90 min incubation. Sodium caprate also resulted in an enhanced P_app_. The greatest absorption of berberine in the presence of sodium caprate, was in the ileum (ER = 3.49), which showed the weakest absorption in the absence of sodium caprate. The duodenum and jejunum showed enhancement ratios (ERs) of 2.08 and 1.49, respectively. In vivo studies demonstrated that the bioavailability of berberine could be improved by co-administration of sodium caprate. A significant increase in the peak plasma concentration (from 721.39 ± 53.46 to 988.84 ± 135.56 ng/mL) of berberine in rats was observed, together with delayed peak time (from 30 to 60 min) and increased AUC_0-6 h_ (28%). Compared with the berberine treatment group, berberine with sodium caprate remarkably decreased the blood glucose levels and the areas under the glucose curves, thus enhancing the antidiabetic activity of insulin [101]. It is suggested that the low bioavailability of berberine is due to P-gp efflux. It has previously been demonstrated that sodium caprate can inhibit P-gp efflux [215].

### 4.25. Zonula Occludens Toxin (Zot)

Research demonstrated that the permeability of high molecular weight markers and poorly bioavailable compounds can be increased across Caco-2 cell monolayers by co-administration of the absorption enhancer Zonula occludens toxin (Zot, MW: 45 kDa), a toxin produced by the *Vibrio cholerae* bacteria [107]. During this study, the transport of hydrophilic high molecular weight markers (i.e., PEG4000, FITC–dextran 10,000, and inulin) and hydrophobic therapeutic agents (i.e., acyclovir, cyclopsorin, paclitaxel, and doxorubicin) were evaluated with Zot using Caco-2 cell monolayers. It has previously been established that Zot can reversibly open tight junctions between intestinal epithelial cells by binding to a specific receptor on the luminal surface of the intestine [216]. After cell monolayers were pre-incubated with Zot (0, 1, 2, and 4 µg/mL) for 30 min, markers or therapeutic agents were added to inserts at time 0 and samples were collected over 120 min from the basolateral chamber. Radio-graphic or HPLC methods were used for transport analysis, and TEER values were monitored over a 3 h period. Results indicated that 4 µg/mL Zot significantly enhanced the transport of markers with MW < 5 kDa by 6.2-fold. Similarly, Zot significantly increased transport of therapeutic agents at a concentration of 4 µg/mL, yielding enhancement ratios of 1.8 and 3.13 for acyclovir and paclitaxel, respectively. Significantly lower TEER values were observed between 0.5 and 2 h, although this effect of Zot on TEER across Caco-2 cell monolayers was reversible.

Caco-2 transport studies with the transcellular marker propranolol indicated that Zot does not significantly modulate the transcellular pathway [107]. Furthermore, a lactate dehydrogenase (LDH) assay demonstrated no significant difference in LDH activity in the presence of Zot, thus suggesting Zot is non-cytotoxic at the effective concentration level after a 3 h incubation period [107]. The permeation of FITC was not significantly enhanced, whereas a significant increase (6.3-fold) in permeability of inulin from 7 × 10^−7^ cm/s (control) to 4.37 × 10^−6^ cm/s (4 µg/mL) was observed. It was suggested that the compact cylindrical configuration of inulin results in higher permeability of inulin vs. PEG4000, even though inulin has a higher molecular weight [107]. The in vitro results with the therapeutic agents displayed a range of permeation increase by Zot from 20–300%. The rank order of permeability enhancement observed with the therapeutic agents in the presence of Zot was paclitaxel > doxorubicin > acyclovir > cyclosporine A. Paclitaxel and cyclosporine A demonstrated P_app_ enhancement ratios of 3.13 and 1.2, respectively [107].

## 5. Pulmonary Route of Administration

**Figure 4:**
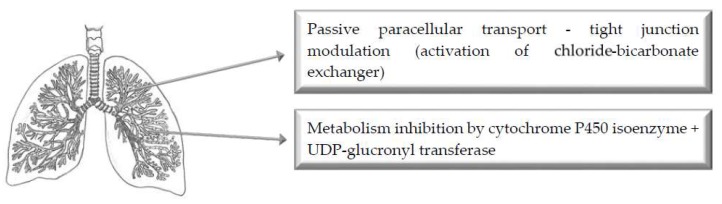
Illustration of the main mechanisms of action of bioenhancers for enhanced pulmonary drug delivery.

Figure 4 illustrates the main mechanisms of action of selected bioenhancers for improved drug delivery via the pulmonary route of administration.

### 5.1. Bile Salts

A research study demonstrated that the tracheal permeability of thyrotropin-releasing hormone (TRH, MW: 362 Da) and insulin (MW: 5814 Da) was significantly increased by sodium glycocholate [113]. Non-everted sac segments of excised rabbit trachea were used. In addition to the transport study, the peptidase activities in the trachea and jejunum were measured and compared to examine the enzymatic barrier for TRH and insulin permeation across rabbit tracheal epithelium [113]. Results demonstrated that the permeability of both TRH (5 mM) and insulin (10 IU/mL) significantly increased in the presence of 10 mM glycocholate. The P_app_ values for TRH (5 and 10 mM) were 2.51 ± 0.31 × 10^−7^ and 3.54 ± 0.87 × 10^−7^ cm/s, respectively. Sodium glycocholate (10 mM) increased the tracheal permeability of TRH ~3-fold [113]. The P_app_ value for insulin (10 IU/mL) was 6.66 ± 0.11 × 10^−9^ cm/s. However, the half-life of insulin was about 14 h, and only showed slight degradation by luminal secreted enzymes for the duration (150 min) of the experiment. The tracheal and jejunal peptidases showed the following decreasing order of activity: DPP IV > Leu-aminopeptidase > cathepsin-B > trypsin [113]. These four peptidases had significantly lower activities in the tracheal epithelial cells compared to jejunal epithelial cells. Since TRH had no metabolites during tracheal permeation, a potential action mechanism of tight junction modulation and therefore enhanced paracellular permeability is suggested for sodium glycocholate [113]. Glycocholate is known to be a Leu-aminopeptidase inhibitor. However, insulin was slightly degraded during tracheal permeation. It is therefore suggested that insulin (MW: 5814 Da) is mainly transported via paracellular diffusion with slight metabolism by proteolytic enzymes such as trypsin-like and aminopeptidase-like enzymes through isolated rabbit tracheal epithelium [113].

Similarly, previous in vitro and in vivo research indicated that absorption of inhaled insulin could be enhanced by the bile salt sodium taurocholate [114]. For the in vivo study, beagle dogs were starved for at least 16 h, after which they were intubated and exposed to insulin or insulin-sodium taurocholate aerosols for differing times. As an intravenous reference, insulin in 0.9% NaCl was infused for 5 min (0.2 U/kg, 0.5 U/mL, 0.08 mL/kg per min) in the right foreleg vein. The insulin–sodium taurocholate solutions were aerosolized by a PARI LC jet nebulizer. The flow from the nebulizer was 3.2 L/min (1 Bar) and the target inhaled dose of insulin was 1 U/kg. Blood samples were collected before dosing and at 5, 10, 15, 25, 35, 50, 65, 95, 125 and 245 min after start (*t* = 0) of inhalation [114]. The bioavailability of pure insulin was 2.6 ± 3%, while the bioavailability of nebulized insulin solutions increased to 23.2 ± 4.4% with addition of 32 mM sodium taurocholate. In comparison, a 3.81 ± 1.12% bioavailability of insulin was obtained when aerosolized powder was administered to the lungs. In vitro results indicated increased insulin transport, accompanied by reduced TEER, at sodium taurocholate concentrations between 25 and 30 mM. Unfortunately, the viability of cell layers was approximately zero at sodium taurocholate concentrations exceeding 32 mM [114].

### 5.2. Chitosan and Derivatives

The enhancement of bronchial octreotide absorption by chitosan and N-trimethyl chitosan (TMC) was evaluated with an integral in vitro/in vivo correlation approach [109]. The TMC derivatives with 20% and 60% QD (TMC20 and TMC60) were synthesized by alkaline methylation of chitosan. Chitosan was dissolved at 1.5% (*w/v*) in HBSS, buffered with 30 mM HEPES at pH 5.5. TMC20 and TMC60 were dissolved at 1.5% (*w/v*) in HBSS/HEPES at pH 7.4. Two hours prior to the TEER studies, the culture medium was removed and Calu-3 cells were equilibrated in 1 ml HBSS/HEPES at pH 7.4 in the basolateral chamber and 200 µL HBSS/HEPES, at pH 5.5 or pH 7.4, in the apical compartment. At *t* = 0, the apical medium was replaced by 200 µL chitosan, TMC20 or TMC60 formulations or by 200 µL HBSS/HEPES at pH 5.5 or pH 7.4 that served as controls. The TEER was measured at *t* = 120 and 60 before administration and *t* = 0, 30, 60, 90, 120, 150, 210 and 240 min after administration.

Octreotide was dissolved to an end concentration of 0.97 mM in 0.9% saline containing 1.5% chitosan (pH 5.5), TMC20 (pH 7.4) or TMC60 (pH 7.4). Control solutions contained octreotide in 0.9% saline of pH 5.5 and 7.4. From these solutions, 200 µL were instilled into the trachea of rats. Intratracheal instillation was employed to ensure dose deposition in bronchial region of rats. Blood samples of 200 µL were collected initially, and every 30 min thereafter for 4 h. A group of 6 rats received a 200 µL intravenous bolus containing 190 µM octreotide in 0.9% saline in order to determine the absolute bioavailability of octreotide [109].

A significant, but reversible, reduction in TEER was observed in the presence of chitosan, TMC20 and TMC60, accompanied by 21-, 16- and 30-fold enhanced octreotide permeation, respectively. The bioavailability was enhanced by 2.4-, 2.5- and 3.9-fold, respectively. TMC60 induced a strong decrease in TEER, which suggested that pH, solubility and cationic charge density are important factors for paracellular barrier modulation [109]. A linear in vitro/in vivo correlation was observed between calculated absorption rates (*R*^2^ = 0.93), which suggested that an analogous mechanism causes permeation enhancement by the polymers, both in vitro and in vivo. The permeation studies showed zero order kinetic profiles, suggesting that the polymers altered the structural integrity of tight junctions to facilitate passive paracellular diffusion of octreotide [109].

### 5.3. Citric Acid (Chelating Agent)

The effect of additives on the pulmonary absorption of insulin from solutions and dry powders was examined in male Sprague–Dawley rats [110]. Results from this study demonstrated a slight hypoglycemic effect in the absence of citric acid [110]. In the presence of citric acid, a significant and extended dose-dependent hypoglycemic effect was observed after insulin administration. Bioavailabilities of 43% and 57% were obtained with the citrate buffer solutions (0.19 mg/dose of citric acid in 0.1 mL) at pH 5 and 3, respectively. However, a greater hypoglycemic effect was achieved with citrate in dry powder than in solution. For the 0.2% (0.036 mg/dose) citric acid formulation, the blood glucose level rapidly decreased after insulin dry powder administration and the effect continued for the duration of the experiment [110]. An absolute bioavailability greater than 50% was achieved. LDH activity, which is a sensitive indicator of acute toxicity to lung cells, was as low for 0.2% citric acid as it was for PBS administration. This suggested that citric acid is a safe absorption enhancing additive. Possible action mechanisms proposed for citric acid included altered epithelium membrane integrity of the lungs, decreased insulin degradation by suppressing enzyme activity and phagocytic activity of alveolar macrophages [110].

### 5.4. Cyclodextrins (CDs)

An in vitro permeability study of chemically modified cyclodextrins (CDs), namely hydroxypropyl-β-cyclodextrin (HPβCD), hydroxypropyl-γ-cyclodextrin (HPγCD), randomly methylated β-cyclodextrin (Rameb), and 2-0-methyl-β-cyclodextrin (Crysmeb), was conducted using Calu-3 cells [111]. Results from this study showed a concentration-dependent increase in ^14^C-mannitol flux across Calu-3 layers in the presence of each of the βCD derivatives, while no change in permeability was caused by HPγCD. Rameb was the only CD that enhanced mannitol transport at the lowest concentration (10 mM), whilst increasing mannitol flux 10-fold at the highest concentration (50 mM). Crysmeb and HPβCD were able to increase mannitol permeability at 25 and 50 mM. A reduction in TEER was observed for all increases in mannitol transport across the Calu-3 cell layers. Confocal microscopy revealed that Crysmeb (25 mM) was able to reversibly disrupt the tight junctional complexes [111].

Similarly, a previous study demonstrated that tetradecyl-β-maltoside (TDM) and dimethyl-β-cyclodextrin (DMβCD) could enhance the pulmonary absorption of low molecular weight heparin (LMWH) both in vitro and in vivo, mainly by acting on the membrane rather than interacting with the drug [115]. Calu-3 cells were used to conduct transport studies with ^3^H-enoxaparin and ^14^C-mannitol with or without different concentrations (0.0625%, 0.125%, and 0.25% (*w/v*)) of TDM or DMβCD at pH 7.4. TEER was measured during the transport experiments. For the in vivo pulmonary absorption studies, TDM and DMβCD were dissolved in saline to prepare 1% stock solutions of each. Enoxaparin, dalteparin (MW: 5000 Da), and unfractionated heparin (MW: 15,000–20,000 Da) were respectively mixed with either saline or TDM and DMβCD (0.0625%, 0.125%, and 0.25% (*w/v*)) to yield a final concentration of 15 U of anti-factor Xa activity per 100 µL formulation. These formulations (50 U/kg) were intratracheally or subcutaneously administered to rats for the absorption and bioavailability studies, respectively. Subsequently, blood samples (300 µL) were collected at 0, 15, 30, 60, 120, 240, 360, and 480 min. Enoxaparin absorption was determined by measuring plasma anti-factor Xa levels using a colorimetric assay, followed by standard pharmacokinetic analysis [115].

Results demonstrated a dose-dependent increase in enoxaparin in the presence of TDM or DMCD (0.0625–0.25%) in the apical fluid. With an increase in TDM concentration from 0.0625% to 0.25%, a statistically significant 4-fold increase in the P_app_ of enoxaparin was observed. Similar results were obtained with the transport of mannitol. Increase in the overall permeability of mannitol in the presence of TDM or DMβCD suggest that the action mechanism is loosening of tight junctions and therefore enhanced paracellular transport. This was confirmed with TEER values that were decreased to 51.9% and 70.7% of the initial value in the presence of 0.25% TDM and 0.25% DMβCD, respectively, after 2 h. However, the effect on TEER reduction was reversible, indicating that these bioenhancers are not likely to cause extensive damage or cellular toxicity in respiratory epithelial cells. Increased concentrations of TDM and DMβCD resulted in increased *C*_max_ and decreased *T*_max_ for enoxaparin. Altogether, results from the pharmacokinetic analysis suggested that TDM enhanced pulmonary absorption of enoxaparin. Bioavailability data showed that TDM was more efficacious in enhancing pulmonary absorption of LMWH when compared to DMβCD. Interestingly, pharmacokinetic profiles of pulmonary administered enoxaparin showed a quicker onset of anti-factor Xa activity compared to subcutaneous enoxaparin. Dalteparin also increased the anti-factor Xa levels rapidly and substantially in the presence of 0.125% TDM or DMβCD, whereas unfractionated heparin only produced a modest increase in anti-factor Xa activity. Reduced absorption of unfractionated heparin was observed, which could be due to the fact that it is larger and bulkier than LMWHs (enoxaparin and dalteparin). Results from this study are in agreement with previous studies on the nasal delivery of LMWHs [217]. Furthermore, comparative analysis demonstrated that the relative bioavailability of enoxaparin was 4- to 6-fold higher with pulmonary administration compared to nasally administered enoxaparin, and the amounts of bioenhancers required to produce therapeutic anti-factor Xa activity from pulmonary administered LMWHs were 2-5 times less [115].

### 5.5. Lanthanides

The absorption of insulin pre-administered or co-administered with lanthanides (Ln^3+^), namely lanthanum, cerium and gadolinium, from rat lung was investigated by means of an in situ pulmonary absorption experiment [112]. Lanthanide ion solutions were prepared by dissolving the oxide in 5 mol/L HCl solution, followed by heating to deplete excess acid. Residue was then diluted with saline water and Ln^3+^ concentrations were determined. For pre-administered intratracheal drug delivery, Ln^3+^ (0.2 mg/kg) in 25 µL saline water (pH 7) was first injected, followed by insulin (1 IU/kg in 40 µL saline water, pH 7) administration 30 min later. In the other experiments, insulin and Ln^3+^ were co-administered. Blood samples (0.5 mL) were collected at predetermined time intervals after insulin administration, and serum insulin levels were measured [112].

Results demonstrated that serum insulin levels increased significantly in the presence of CeCl_3_ and GdCl_3_, with relative bioavailabilities of 57.9% and 59.5%, respectively. Furthermore, the anionic form of gadolinium showed greater enhancement of pulmonary insulin absorption than its cationic form (Gd^3+^). The relative bioavailabilities of co-administered and pre-administered GdCl_3_ were 80.1% and 59.5%, respectively. In comparison, LaCl_3_ showed weak enhancement in pulmonary absorption of insulin, with a relative bioavailability of 30.9%, while LuCl_3_ had no enhancing effect [112]. Co-administration of Gd^3+^ (0.2 mg/kg) with insulin (Fr = 80.1%) showed the greatest insulin absorption enhancement from the lung. Interestingly, higher concentrations of Gd^3+^ (0.6 mg/kg) showed decreased insulin absorption, possibly due to partial hydrolysis of Gd^3+^ upon entrance of the lung. The action mechanism proposed for Ln^3+^ is local mucosal tissue modulation, whereby lanthanides bind to membrane lipids and proteins to induce conformational changes and micropore formation to enhance the permeability of intracellular and exogenous matter flux [218,219]. Another action mechanism is the favorable absorption of insulin from the lung when a conformational change of insulin is induced when Ln^3+^ binds to zinc(II) binding sites. Additionally, increased interaction of insulin with its receptor in the cell membrane might be increased with insulin-bound Ln^3+^ in the serum [112].

### 5.6. Protease Inhibitors

The effects of protease inhibitors were evaluated as absorption enhancers for the pulmonary absorption of recombinant human colony-stimulating factor (rhG-CSF) in rats [108]. Protease inhibitors included (*p*-amidinophenyl) methanesulfonyl fluoride-HCl (*p*-APMSF), aprotinin and bestatin. The respective protease inhibitors (10 mM *p*-APMSF, 500 IU/mL aprotinin, 1 mM bestatin) were added to rhG-CSF solution (pH 6.5), after which the final rhG-CSF concentration was adjusted to 250 µg/mL. After intravenous, subcutaneous and intratracheal administration of the test solutions (containing protease inhibitors) and the control solution (rhG-CSF only), blood samples were collected periodically for 8h to determine the rhG-CSF plasma concentration by enzyme immunoassay.

The plasma rhG-CSF concentration was greatly enhanced in the presence of protease inhibitors, with *p*-APMSF showing the greatest enhancement with a ~3-fold increase in plasma rhG-CSF concentration from 3.9 to 11.7 ng/mL 30 min after intratracheal administration [108]. In order to determine the mechanism of enhanced absorption, a surfactant, polyoxyethylene 9-lauryl ether (Laureth-9) and the protease inhibitor *p*-APMSF were administered with rhG-CSF. The rhG-CSF concentration increased ~123-fold from 3.9 ± 1.4 ng/mL to 481.5 ± 96.7 ng/mL with the simultaneous intratracheal administration of Laureth-9 and *p*-APMSF. As a result, the proposed mechanism of action entails enhanced membrane permeation and inhibition of enzymatic degradation of rhG-CSF [108].

Similarly, a previous study demonstrated that the tracheal permeability of insulin was significantly increased by bestatin (aminopeptidase B and leucine aminopeptidase inhibitor), and aprotinin (trypsin and chymotrypsin inhibitor) [113]. Non-everted sac (2 cm) segments of excised rabbit trachea were used. Briefly, test solutions, with or without protease inhibitors (0.1 and 1 mM bestatin; 1000 and 10,000 KIU/mL aprotinin), in HEPES-buffered solution (0.2 mL, pH 7.4) was infused into the sac mucosal side, which was then placed in serosal medium consisting of oxygenated (O_2_/CO_2_, 95%:5%) HEPES (7 mL, pH 7.4). Samples (0.2 mL) were removed from the serosal fluid at pre-determined times for 150 min, and replaced with 0.2 mL fresh fluid. In another experiment, 10 µL samples were removed from the mucosal fluid at pre-determined times to calculate the degradation rate of insulin in the mucosal fluid of the trachea using the RIA method. Additionally, the peptidase activities in the trachea and jejunum were measured and compared to examine the enzymatic barrier for TRH and insulin permeation across rabbit tracheal epithelium [113].

Results demonstrated that the permeability of insulin (10 IU/mL) significantly increased in the presence of 1 mM bestatin and 10,000 KIU/mL aprotinin. However, the half-life of insulin was about 14 h, and only showed slight degradation by luminal secreted enzymes for the duration (150 min) of the experiment [113]. The tracheal and jejunal peptidases showed the following decreasing order of activity: DPP IV > Leu-aminopeptidase > cathepsin-B > trypsin. These four peptidases had significantly lower activities in the tracheal epithelial cells compared to jejunal epithelial cells. However, insulin was slightly degraded during tracheal permeation. It is suggested that insulin (MW: 5814 Da) is mainly transported via paracellular diffusion with slight metabolism by proteolytic enzymes such as trypsin-like and aminopeptidase-like enzymes through isolated rabbit tracheal epithelium. In summary, pulmonary administration of some peptide drugs, such as insulin, by intratracheal insufflation and instillation may contribute to the systemic absorption of these drugs [113].

### 5.7. Surfactants

The effects of surfactants of natural origin were evaluated as absorption enhancers for the pulmonary absorption of recombinant human colony-stimulating factor (rhG-CSF) in rats [108]. Surfactants included polyoxyethylene 9-lauryl ether (Laureth-9) and sodium glycocholate (SGC). Surfactants (1% *w/v*) were respectively added to rhG-CSF solution (pH 6.5), after which the final rhG-CSF concentration was adjusted to 250 µg/mL. After intravenous and intratracheal administration of the test solutions (containing surfactants) and the control solution (rhG-CSF only), blood samples were collected periodically for 8 h to determine the rhG-CSF plasma concentration by enzyme immunoassay.

The plasma rhG-CSF concentration was greatly enhanced in the presence of the surfactants. Relative bioavailabilities of rhG-CSF achieved with intravenous and intratracheal administration of Laureth-9 and SGC were 37% (intravenous), 88% (intratracheal), 84% (intravenous), and 197% (intratracheal), respectively [108]. In order to determine the mechanism of enhanced absorption, both the surfactant Laureth-9 and a protease inhibitor (*p*-amidinophenyl) methanesulfonyl fluoride-HCl (*p*-APMSF) were administered with rhG-CSF. The rhG-CSF concentration increased ~123-fold from 3.9 ± 1.4 ng/mL to 481.5 ± 96.7 ng/mL with the simultaneous intratracheal administration of Laureth-9 and *p*-APMSF. As a result, the proposed mechanism of action entails enhanced membrane permeation and inhibition of enzymatic degradation of rhG-CSF [108].

## 6. Conclusions

A comprehensive list of bioenhancers of natural origin is given in Table 1 with their mechanisms of action for four specific routes of drug administration, namely nasal, buccal, pulmonary, and oral. From all the described bioenhancing studies it is evident that inhibition of pre-systemic metabolism as well as efflux transporters and tight junction modulation is the predominant mechanism of drug bioavailability enhancement for the oral route of drug administration. Tight junction modulation and membrane disruption are the pre-dominant mechanisms of drug absorption enhancement for the nasal, buccal and pulmonary routes of administration. Since various factors can influence dissolution and absorption of drugs across different mucosal surfaces, studies on bioenhancers should be conducted for each specific drug for which bioavailability enhancement is needed. Although many studies have shown the potential of absorption enhancing agents, very few have been included in commercially available drugs.

## Figures and Tables

**Table 1:** Summary of selected natural bioenhancers and their main mechanisms of action on various drugs for enhanced nasal, oral, buccal and pulmonary drug delivery.

Route of Administration	Bioenhancer (Class)	Biological Source	Mechanism(s) of Action	Study Design Model	Research Compound	Reference(s)
Buccal	*Aloe vera* (gel, whole leaf)	Plant (*Aloe vera*)	Intercellular modulation	In vitro (Franz diffusion cells)	Didanosine: Antiviral reverse transcriptase inhibitor	[20]
Buccal	Chitosan (Biopolymer)	Deacetylated chitin from crustaceans and fungi	Mucoadhesion; changes in lipid organization and loosening of intercellular filaments	In vitro (T146 cells ^1^)	FITC–dextran: Hydrophilic polysaccharide	[21]
Buccal	Chitosan (Biopolymer)	Deacetylated chitin from crustaceans and fungi	Mucoadhesion; mucosal membrane modulation	Ex vivo (porcine buccal mucosa)	Hydrocortisone: Corticosteroid TGF-beta: Cytokine polypeptide	[22]
Buccal	Chitosan–TBA (Thiolated polymer)	Deacetylated chitin from crustaceans and fungi	Mucoadhesion; mucosal membrane modulation	Ex vivo (porcine buccal mucosa); in vivo (pig)	PACAP: Pituitary Adenylate Cyclase-activating Peptide	[23,24]
Buccal	Cod-liver oil extract (Fatty acid)	Animal (Cod fish)	No mechanism specified	Ex vivo (hamster cheek pouch)	Ergotamine tartrate: Ergopeptine alkaloid	[25]
Buccal	Menthol (Alcohol)	Plant (Corn mint, peppermint, or other mint oils)	No mechanism specified	Ex vivo (porcine buccal mucosa)	Dideoxycytidine: Nucleoside analog reverse transcriptase inhibitor (NRTI)	[26]
Buccal	Oleic acid, eicosapentaenoic acid, docosahexaenoic acid (Fatty acids)	Animal (Cod fish)	No mechanism specified	In vitro (membraneless dissolution test), in vivo (rat)	Insulin: Peptide hormone	[27]
Buccal	Sodium glycodeoxycholate (Bile salt)	Intestinal bacterial by-product	No mechanism specified	Ex vivo (porcine buccal mucosa)	Dideoxycytidine: Nucleoside analog reverse transcriptase inhibitor (NRTI)	[28]
Buccal	TMC (Cationic polymers)	Chemically modified chitosan (crustaceans, fungi)	Mucoadhesion; mucosal membrane modulation	Ex vivo (porcine buccal mucosa)	FD4: Hydrophilic polysaccharide	[29]
Nasal	Chitosan (Biopolymer)	Chemically modified chitosan (crustaceans, fungi)	Tight junction modulation	In vivo (sheep)	sCT: Endogenous polypeptide hormone	[30]
Nasal	Chitosan (Biopolymer)	Deacetylated chitin from crustaceans and fungi	Increased mucoadhesion; tight junction modulation	In vivo (sheep, human)	Morphine: Opium alkaloid	[31]
Nasal	Chitosan–TBA (Thiolated polymer)	Deacetylated chitin from crustaceans and fungi	Increased mucoadhesion; tight junction modulation	In vivo (rat)	Insulin: Peptide hormone	[32]
Nasal	TMC (Cationic polymers)	Chemically modified chitosan (crustaceans and fungi)	Increased mucoadhesion; tight junction modulation	In vivo (rat)	Mannitol: Sugar alcohol	[33]
Oral	(-)-Epicatechin (Flavonoid)	Plant (woody plants)	Metabolism (glucuronidation) inhibition	Ex vivo (rat small intestine)	Alpha-naphtol: Organic fluorescent compound	[34]
Oral	*Aloe vera* (gel and whole leaf)	Plant (*Aloe vera*)	Tight junction modulation	Ex vivo (rat intestinal tissue)	Atenolol: Beta-receptor activity compound	[35]
Oral	Aloe vera (gel and whole leaf)	Plant (*Aloe vera*)	Tight junction modulation	In vitro (Caco-2 cells ^2^)	Insulin: Peptide hormone	[36]
Oral	Aloe vera (juice)	Plant (*Aloe vera*)	Local mucosal tissue modulation	In vivo (human)	Vitamin C and E: Ascorbic acid, tocopherols, tocotrienols.	[37]
Oral	Aloe vera (gel polysaccharides)	Plant (*Aloe vera*)	Metabolism inhibition; tight junction modulation	In vitro (Caco-2, LS180 cells ^3^), In vivo (rat)	Indinavir: Antiviral protease inhibitor	[38]
Oral	BHCl (Flavonoid)	Acidification of betaine Plant (beetroot: *Beta vulgaris*)	Metabolism enhancement (transient re-acidification of gastric pH)	In vivo (human)	Dasatinib: Protein kinase inhibitor	[39]
Oral	Caraway (Flavonoid)	Plant (meridian fennel/Persian cumin: *Carum carvi*)	Local mucosal tissue modulation	In vivo (human)	Rifampicin: Semisynthetic rifamycin derivative, Isoniazid: Isonicotinic acid derivative, pyrazinamide: nicotinamide pyrazine analogue	[40]
Oral	Chitosan (Biopolymer)	Deacetylated chitin from crustaceans and fungi	Tight junction modulation	In vitro (HT-29 clone B6 cells ^4^)	Heparin: Anticoagulant	[41]
Oral	Chitosan (Biopolymer)	Deacetylated chitin from crustaceans and fungi	Tight junction modulation	In vitro (Caco-2 cells ^2^)	Chitosan– (Lissamine–rhodamine labelled)	[42]
Oral	Chitosan–TBA (Thiolated polymer)	Deacetylated chitin from crustaceans and fungi	Mucoadhesion; tight junction modulation	Ex vivo (guinea pig small intestinal mucosa)	Cefadroxil: Cephalosporin	[43]
Oral	Chitosan–TBA (Thiolated polymer)	Deacetylated chitin from crustaceans and fungi	Mucoadhesion; tight junction modulation	In vivo (rat)	Insulin: Peptide hormone	[44]
Oral	Curcumin (Flavonoid)	Plant (turmeric: *Curcuma longa*)	Metabolism (UDP-glucuronyl transferase) inhibition	In vitro (rat microsomes)	Mycohenolic acid: Immunosuppressant	[45]
Oral	Curcumin (Flavonoid)	Plant (turmeric: *Curcuma longa*)	Efflux transporter inhibition; metabolism inhibition	In vivo (rabbit)	Norfloxacin: Fluoroquinolone	[46]
Oral	Curcumin (Flavonoid)	Plant (turmeric: *Curcuma longa*)	Metabolism (CYP3A4) inhibition	In vitro (human liver microsomes)	Midazolam: Benzodiazepine	[47]
Oral	Curcumin (Flavonoid)	Plant (turmeric: *Curcuma longa*)	Efflux transporter (P-gp) inhibition; metabolism (CYP3A4) inhibition	In vivo (rat)	Midazolam: Benzodiazepine	[48]
Oral	Cyclosporine A (Immunosuppressant)	Fungi (*Tolypocladium inflatum Gams*)	Efflux transporter (P-gp) inhibition	In vivo (rat, dog)	Clopidogrel: Platelet aggregation inhibitor	[49]
Oral	Diosmin (Flavonoid)	Plant (citrus fruits)	Efflux transporter (P-gp) inhibition	In vitro (Caco-2 cells ^2^)	Digoxin: Digitalis glycoside	[50]
Oral	Emodin (Anthraquinone derivative)	Plant (senna: *Cassia angustifolia*, *Aloe vera* (syn *Aloe barbadensis*), rhubarb: *Rheum officinale*)	Efflux transporter (P-gp) inhibition	In vitro (MDR1-MDCKII cells ^6^, Caco-2 cells^2^)	Digoxin: Digitalis glycoside	[51]
Oral	Fulvic acid (Organic acid)	Plant (decomposed material)	Metabolism enhancement (enhanced drug water solubility)	In vivo (rat)	Glibenclamide: Sulfonylurea antidiabetic Insulin: Peptide hormone Pentazocin: Opioid analgesic	[52]
Oral	Gallic acid ester (Organic acid)	Plant (gallnuts, sumac, witch hazel, tea leaves, oak bark)	Metabolism (CYP3A) inhibition	In vitro (human liver microsomes)	Nifedipine: Calcium channel blocker	[53]
Oral	Genistein (Flavonoid)	Plant (soyabean: *Glycine max, kudzu: Pueraria lobata*)	Efflux transporter (MRP) inhibition	In vitro (HT-29 cells^4^), In vivo (rat)	Epigalllocatechin-3-gallate (EGCG): Phenolic antioxidant	[54]
Oral	Genistein (Flavonoid)	Plant (soyabean: *Glycine max, kudzu: Pueraria lobata*)	Efflux transporter (P-gp, BCRP, MRP2) inhibition; metabolism (CYP3A4) inhibition	In vivo (rat)	Paclitaxel: Tetracyclic diterpenoid	[55]
Oral	Gokhru extract (Herbal)	Plant (Tribulus: *Tribulus terrestris*)	Local mucosal tissue modulation	In vitro (goat everted sac)	Metformin: Biguanide	[56]
Oral	Gokhru extract (Herbal)	Plant (Tribulus: *Tribulus terrestris*)	Local mucosal tissue modulation	In vitro (chicken everted intestine)	Metformin: Biguanide	[57]
Oral	Grapefruit juice (Citrus fruit)	Plant (grapefruit: *Citrus paradisi*)	Efflux transporter (P-gp, MRP2); metabolism (CYP3A4) inhibition; renal uptake transporter (OATP) inhibition	Various	Various	[58]
Oral	LSC (Chitosan derivative)	Modified chitosan (crustaceans and fungi)	Increased mucoadhesion; tight junction modulation	In vitro (Caco-2 cells ^2^), In vivo (rat), Ex vivo (rat intestine)	Insulin: Peptide hormone	[59]
Oral	Lycopene (Carotenoid)	Plant (red fruits and vegetables)	Dual carotenoid/LDL receptor mechanism for targeted hepatic delivery	In vivo (human)	Simvastatin: HMG–CoA reductase inhibitor	[60]
Oral	Lysergol (Alkaloid)	Plant (morning glory plant: *Ipomoea* spp.)	Metabolism inhibition	In vivo (rat)	Berberine: Benzylisoquinoline alkaloid	[61]
Oral	Lysergol (Alkaloid)	Plant (morning glory plant: *Ipomoea* spp.)	Efflux transporter (BCRP) inhibition; metabolism inhibition	In vitro (rat liver microsomes)	Curcumin: Zingiberaceae Sulfasalazine: Aminosalicylic agent	[62]
Oral	Moringa oleifera pods (Traditional herbal medicine)	Plant (*Moringa oleifera*)	Metabolism (CYP450) inhibition	In vivo (mice)	Rifampicin: Semisynthetic rifamycin derivative	[63]
Oral	Naringin (Flavonoid glycoside)	Plant (grapefruit, apple, onion, tea)	Efflux transporter (P-gp) inhibition; metabolism inhibition	In vivo (rat)	Diltiazem: Benzothiazepine derivates	[64]
Oral	Naringin (Flavonoid glycoside)	Plant (grapefruit, apple, onion, tea)	Metabolism (CYP3A4) inhibition	In vivo (rat)	Tamoxifen: selective estrogen receptor modulator (SERM)	[65]
Oral	Naringin (Flavonoid glycoside)	Plant (grapefruit, apple, onion, tea)	Efflux transporter (P-gp) inhibition; metabolism (CYP3A4) inhibition	In vivo (rat)	Paclitaxel: Tetracyclic diterpenoid	[66]
Oral	Naringin (Flavonoid glycoside)	Plant (grapefruit, apple, onion, tea)	Efflux transporter (P-gp) inhibition; metabolism (CYP3A4) inhibition	Ex vivo (rat everted gut sac)	Clopidogrel: Platelet aggregation inhibitor	[67]
Oral	Naringin (Flavonoid glycoside)	Plant (grapefruit, apple, onion, tea)	Metabolism (CYP3A4) inhibition	In vivo (rabbit)	Verapamil: Calcium channel blocker	[68]
Oral	Palmitoyl carnitine chloride (Chelating agents)	Esterification of carnitinePlant/animal (various)	Tight junction modulation	In vitro (Caco-2 cells ^2^)	Clodronate: Bisphosphonate	[69]
Oral	Peppermint oil (Herbal)	Plant (peppermint: *Mentha pipertita*)	Metabolism (CYP3A) inhibition	Ex vivo (rat intestinal tissue)	Cyclosporine: Immunosuppressant	[70]
Oral	Piperine (Alkaloid)	Plant (*Piper longum* and *Piper nigrum*)	Local mucosal tissue modulation; thermogenic activity	In vivo (human)	B-carotene: Terpenoid	[71]
Oral	Piperine (Alkaloid)	Plant (*Piper longum* and *Piper nigrum*)	Local mucosal tissue modulation; thermogenic activity	In vivo (human)	Coenzyme Q10: benzoquinone	[72]
Oral	Piperine (Alkaloid)	Plant (*Piper longum* and *Piper nigrum*)	Decreased elimination (gastrointestinal transit inhibition; gastric emptying inhibition)	In vivo (rat, mice)	Phenol red: Spheroid	[73]
Oral	Piperine (Alkaloid)	Plant (*Piper longum* and *Piper nigrum*)	Metabolism inhibition	In vivo (human)	Propanol: Beta-receptor activity compound, theophylline: methylxanthine	[74]
Oral	Piperine (Alkaloid)	Plant (*Piper longum* and *Piper nigrum*)	Metabolism (CYP450) inhibition	In vivo (rat)	Nimesulide: Non-steroidal anti-inflammatory	[75]
Oral	Piperine (Alkaloid)	Plant (*Piper longum* and *Piper nigrum*)	Efflux transporter (P-gp) inhibition	In vivo (rat)	Fexofenadine: Terfenadine metabolite	[76]
Oral	Piperine (Alkaloid)	Plant (*Piper longum* and *Piper nigrum*)	Metabolism inhibition	In vivo (mice)	Resveratrol: Phytoalexin	[77]
Oral	Piperine (Alkaloid)	Plant (*Piper longum* and *Piper nigrum*)	Metabolism inhibition	In vivo (human)	Nevirapine: Non-nucleoside reverse transcriptase inhibitor	[78]
Oral	Piperine (Alkaloid)	Plant (*Piper longum* and *Piper nigrum*)	Metabolism inhibition	In vivo (mice)	Epigalllocatechin-3-gallate (EGCG): Phenolic antioxidant	[79]
Oral	Piperine (Alkaloid)	Plant (*Piper longum* and *Piper nigrum*)	Metabolism inhibition	In vivo (rat)	Pentobarbitone: Barbiturate.	[80]
Oral	Piperine (Alkaloid)	Plant (*Piper longum* and *Piper nigrum*)	Metabolism (CYP3A4) inhibition	In vivo (human)	Carbamazepine: Carboxamide derivative	[81]
Oral	Piperine (Alkaloid)	Plant (*Piper longum* and *Piper nigrum*)	Metabolism (CYP450) inhibition	In vivo (rat)	Nateglinide: Meglitinide	[82]
Oral	Piperine (Alkaloid)	Plant (*Piper longum* and *Piper nigrum*)	Metabolism (hepatic and intestinal glucuronidation) inhibition	In vivo (rat, human)	Curcumin: Zingiberaceae agent	[83]
Oral	Piperine (Alkaloid)	Plant (*Piper longum* and *Piper nigrum*)	Metabolism inhibition	In vivo (hen)	Oxytetracycline: Bacterial protein synthesis inhibitor	[84]
Oral	Quercetin (Flavonoid)	Plant (citrus fruits, vegetables, leaves, grains)	Efflux transporter (P-gp) inhibition	In vivo (rat), Ex vivo (rat and chick everted intestinal sac)	Ranolazine: Piperazine derivative	[85]
Oral	Quercetin (Flavonoid)	Plant (citrus fruits, vegetables, leaves, grains)	Efflux transporter (P-gp) inhibition	In vivo (rat), In vitro (Caco-2 cells ^2^)	Irinotecan: Cytotoxic alkaloid	[86]
Oral	Quercetin (Flavonoid)	Plant (citrus fruits, vegetables, leaves, grains)	Efflux transporter (P-gp) inhibition	In vivo (rats), Ex vivo (rat intestinal everted sac)	Valsartan: Angiotensin II receptor antagonist	[87]
Oral	Quercetin (Flavonoid)	Plant (citrus fruits, vegetables, leaves, grains)	Metabolism (CYP3A) inhibition	In vivo (rabbit)	Verapamil: Calcium channel blocker	[88]
Oral	Quercetin (Flavonoid)	Plant (citrus fruits, vegetables, leaves, grains)	Efflux transporter (P-gp) inhibition; metabolism (CYP3A) inhibition	In vivo (rabbit)	Dilitiazem: Nondihydropyridine calcium channel blocker	[89]
Oral	Quercetin (Flavonoid)	Plant (citrus fruits, vegetables, leaves, grains)	Efflux transporter (P-gp) inhibition; metabolism (CYP3A) inhibition	In vivo (rat)	Doxorubicin: Daunorubicin precursor	[90]
Oral	Quercetin (Flavonoid)	Plant (citrus fruits, vegetables, leaves, grains)	Efflux transporter (P-gp) inhibition	In vivo (human)	Fexofenadine: Terfenadine metabolite	[91]
Oral	Quercetin (Flavonoid)	Plant (citrus fruits, vegetables, leaves, grains)	Efflux transporter (P-gp) inhibition	In vivo (rat, dog)	Clopidogrel: Platelet aggregation inhibitor	[49]
Oral	Quercetin (Flavonoid)	Plant (citrus fruits, vegetables, leaves, grains)	Efflux transporter (P-gp) inhibition; metabolism (CYP3A) inhibition	In vivo (rat)	Etoposide: Podophyllotoxin derivative	[92]
Oral	Quercetin (Flavonoid)	Plant (citrus fruits, vegetables, leaves, grains)	Efflux transporter (P-gp) inhibition; metabolism (CYP3A) inhibition	Various	Epigalllocatechin-3-gallate (EGCG): Phenolic antioxidant	[93]
Oral	Quercetin (Flavonoid)	Plant (citrus fruits, vegetables, leaves, grains)	Efflux transporter (P-gp) inhibition	In vitro (human MCF-7 ADRr cells ^7^)	Doxorubicin: Daunorubicin precursor	[94]
Oral	Quercetin (Flavonoid)	Plant (citrus fruits, vegetables, leaves, grains)	Efflux transporter (MRP) inhibition; metabolism (CYP3A) inhibition	In vivo (rat)	Tamoxifen: selective estrogen receptor modulator (SERM)	[95]
Oral	Quercetin (Flavonoid)	Plant (citrus fruits, vegetables, leaves, grains)	Metabolism (CYP3A) inhibition	In vivo (rat)	Pioglitazone: Thiazolidinedione	[96]
Oral	Quinidine (Class I antiarrhythmic agent)	Chemically modified: stereoisomer of quinine Plant (cinchona tree: *Cinchona* sp.)	Efflux transporter (P-gp) inhibition	Ex vivo (everted rat gut sac)	Paeoniflorin: *Paeonia lactiflora* derivative	[97]
Oral	Resveratrol (Polyphenolic phytoalexin)	Plant (berries, grape skins, red wine)	Metabolism (CYP2C9, CYP2E1) inhibition	In vivo (human)	Diclofenac: NSAID	[98]
Oral	Resveratrol (Polyphenolic phytoalexin)	Plant (berries, grape skins, red wine)	Efflux transporter (P-gp, MRP-2) inhibition; reduced elimination; renal uptake transporter (OAT1, OAT3) inhibition	In vitro (Caco-2 cells ^2^, mock-MDCK, MDR1-MDCK ^6^, MRP2-MDCK ^6^, mock-HEK293, hOAT1-HEK293 ^8^, hOAT3-HEK293 ^8^ cells), Ex vivo (rat everted intestine, rat kidney slices), In vivo (rat)	Methotrexate: Immunosuppressant	[99]
Oral	Sinomenine (Alkaloid)	Plant (*Sinomenium acutum*)	Efflux transporter (P-gp) inhibition	Ex vivo (everted rat gut sac)	Paeoniflorin: *Paeonia lactiflora* derivative	[97]
Oral	Sinomenine (Alkaloid)	Plant (*Sinomenium acutum*)	Efflux transporter (P-gp) inhibition	In vivo (rat)	Paeoniflorin: *Paeonia lactiflora* derivative	[100]
Oral	Sodium caprate (Fatty acid)	Chemically modified: salification of caproic acid Animal (fats and oils)	Tight junction modulation	In situ (recirculating intestinal perfusion), ex vivo (everted rat gut sacs), in vivo (rat)	Berberine: Antidiabetic plant alkaloid	[101]
Oral	Sodium cholate/phospholipid-mixed micelles (Bile salts)	Intestinal bacterial by-product	Mucosal membrane modulation	In vivo (dog)	Silybin, the major active component of silymarin (antihepatotoxic polyphenolic substance isolated from milk thistle plant, Silybum marianum)	[102]
Oral	Soybean phosphotidylcholine/sodium deoxycholate (SPC/SDC) (Bile salts)	SPC: plant (soya bean: *Glycine max*) SDC: chemically modified: salification of deoxycholic acid (metabolic byproduct of intestinal bacteria)	Mucosal membrane modulation	In vivo (dog)	Fenofibrate	[103]
Oral	Tamarixetin (metabolite of quercetin) (Flavonoid)	Plant (hogweed/cow parsnip: *Heracleum stenopterum*)	Metabolism (CYP2C isozyme) inhibition	In vitro (rat liver microsomes), In vivo (rat)	Fluvastatin: HMG CoA reductase inhibitor	[104]
Oral	TMC (Cationic polymers)	Modified chitosan (crustaceans, fungi)	Mucoadhesion; tight junction modulation	In vitro (Caco-2 cells ^2^)	Mannitol: Sugar alcohol PEG 4000: Polyethylene glycol	[105]
Oral	TMC (Cationic polymers)	Modified chitosan (crustaceans, fungi)	Tight junction modulation	In vitro (Caco-2 cells ^2^)	Mannitol: Sugar alcohol FITC–dextran: Hydrophilic polysaccharide Buserelin: Gonadotropin-releasing hormone agonist	[106]
Oral	TMC (Cationic polymers)	Modified chitosan (crustaceans, fungi)	Tight junction modulation	In vitro (Caco-2 cells ^2^)	Clodronate: Bisphosphonate	[69]
Oral	ZOT (Toxins and venom extracts)	Bacteria (*Vibrio cholerae*)	Tight junction modulation	In vitro (Caco-2 cells ^2^)	PEG 4000: Polyethylene glycol FITC–dextran: Hydrophilic polysaccharide Inulin: Naturally occurring polysaccharide Paclitaxel: Tetracyclic diterpenoid Acyclovir: HSV-specified DNA polymerases inhibitor Cyclosporine: Immunosuppressant Doxorubicin: Daunorubicin precursor	[107]
Pulmonary	Aprotinin, bestatin (Protease inhibitors)	Animal (bovine lung tissue), bacteria (*Streptomyces olivoreticuli*)	Metabolism inhibition	In vivo (rat)	rhG-CSF: Granulocyte-colony stimulating factor	[108]
Pulmonary	Chitosan (Biopolymer)	Chemically modified: deacetylation of chitin Animal (crustaceans), fungi	Tight junction modulation	In vitro (Calu-3 cells ^5^); in vivo (rat)	Octreotide: Somatostatin analog	[109]
Pulmonary	Citric acid (Chelating agents)	Plant (citrus fruits and vegetables), fungi (*Aspergillus niger*)	Local mucosal tissue modulation; metabolism inhibition	In vivo (rat)	Insulin: Peptide hormone	[110]
Pulmonary	HPBCD, Crysmeb (Cyclodextrin derivatives)	Plant (starch)	Tight junction modulation	In vitro (Calu-3 cells ^5^)	Mannitol: Sugar alcohol	[111]
Pulmonary	Lanthanum, cerium, gadolinium (Lanthanides)	Natural elements	Drug targeting	In vivo (rat)	Insulin: Peptide hormone	[112]
Pulmonary	Sodium glycocholate (Bile salt)	Intestinal bacterial by-product	Tight junction modulation	Ex vivo (rabbit trachea and jejunum)	Thyrotropin-releasing hormone (TRH): Tripeptidal hypothalamus hormone Insulin: Peptide hormone	[113]
Pulmonary	Sodium taurocholate (Bile salt)	Intestinal bacterial by-product	Metabolism enhancement (dissociation of insulin hexamers); tight junction modulation; metabolism (enzymatic degradation) inhibition	In vitro (Caco-2 cells ^2^), In vivo (dog)	Insulin: Peptide hormone	[114]
Pulmonary	Dideoxycytidine: Nucleoside analog reverse transcriptase inhibitor (NRTI)	Plant (Starch)	Tight junction modulation	In vitro (Calu-3 cells ^5^), in vivo (rat)	Enoxaparin: Anticoagulant	[115]
Pulmonary	TMC (Cationic polymers)	Chemically modified: deacetylation of chitin Animal (crustaceans), fungi	Tight junction modulation	In vitro (Calu-3 cells ^5^); in vivo (rat)	Octreotide: Octapeptide	[109]

^1^ T146 cells: buccal epithelium cells. ^2^ Caco-2 cells: human epithelial colorectal adenocarcinoma cells. ^3^ LS180 cells: intestinal human colon adenocarcinoma cells. ^4^ HT-29 clone B6: human colon carcinoma cells. ^5^ Calu-3 cells: mammalian airway epithelium cells. ^6^ MDR1-MDCKII/MRP2-MDCK cells: Madin–Darby Canine Kidney cells with multidrug resistance 1 (MDR1) or multidrug resistance-associated protein 2 (MRP2) gene. ^7^ MCF-7 ADRr (re-designated NCI-ADR-RES) cells: ovarian tumor cells. ^8^ hOATP1/3-HEK293 cells: human embryonic kidney cells transfected with human organic anion-transporting polypeptide 1 or 3.
